# Opinions about euthanasia and advanced dementia: a qualitative study among Dutch physicians and members of the general public

**DOI:** 10.1186/1472-6939-16-7

**Published:** 2015-01-28

**Authors:** Pauline SC Kouwenhoven, Natasja JH Raijmakers, Johannes JM van Delden, Judith AC Rietjens, Donald G van Tol, Suzanne van de Vathorst, Nienke de Graeff, Heleen AM Weyers, Agnes van der Heide, Ghislaine JMW van Thiel

**Affiliations:** Julius Center for Health Sciences and Primary Care, University Medical Center Utrecht, Room number STR.5.133, Internal post number STR.6.131, P.O. Box 85500, 3508 GA Utrecht, The Netherlands; Department of Public Health, Erasmus Medical Center Rotterdam, Rotterdam, The Netherlands; Department of General Practice, University Medical Center Groningen, Groningen, The Netherlands; Department of Ethics and Philosophy of Medicine, Erasmus Medical Center Rotterdam, Rotterdam, The Netherlands; Department of Legal Theory, University of Groningen, Groningen, The Netherlands

**Keywords:** Euthanasia, Dementia, End-of-life decisions, Public opinion, Ethics, Law

## Abstract

**Background:**

The Dutch law states that a physician may perform euthanasia according to a written advance euthanasia directive (AED) when a patient is incompetent as long as all legal criteria of due care are met. This may also hold for patients with advanced dementia. We investigated the differing opinions of physicians and members of the general public on the acceptability of euthanasia in patients with advanced dementia.

**Methods:**

In this qualitative study, 16 medical specialists, 19 general practitioners, 16 elderly physicians and 16 members of the general public were interviewed and asked for their opinions about a vignette on euthanasia based on an AED in a patient with advanced dementia.

**Results:**

Members of the general public perceived advanced dementia as a debilitating and degrading disease. Physicians emphasized the need for direct communication with the patient when making decisions about euthanasia. Respondent from both groups acknowledged difficulties in the assessment of patients’ autonomous wishes and the unbearableness of their suffering.

**Conclusion:**

Legally, an AED may replace direct communication with patients about their request for euthanasia. In practice, physicians are reluctant to forego adequate verbal communication with the patient because they wish to verify the voluntariness of patients’ request and the unbearableness of suffering. For this reason, the applicability of AEDs in advanced dementia seems limited.

**Electronic supplementary material:**

The online version of this article (doi:10.1186/1472-6939-16-7) contains supplementary material, which is available to authorized users.

## Background

Euthanasia and physician-assisted suicide are permitted by law in the Netherlands, if performed by a physician who meets the six criteria of due care (Table 1) and reported to one of the review committees. Dutch law states that a physician may act according to an advance euthanasia directive (AED) when the patient is incompetent as long as all criteria of due care are met. In this way, the law provides a legal possibility for euthanasia in patients with advanced dementia based on an AED. In 2011, the first publicly known case of euthanasia in a patient with advanced dementia was reported and assessed by a review committee. The review committee judged - after extensive deliberation and consultation - that in this case, the due care criteria were met. However, the Royal Dutch Medical Association issued guidelines in 2010 and 2012 stating that the possibility of direct communication with the patient is indispensable in order to meet the criteria of due care [1, 2]. Therefore, it remains a topic of debate in the Netherlands whether euthanasia in patients with advanced dementia is acceptable [3]. In our study on Knowledge and Opinions of the Public and Professionals regarding End of Life decisions (the KOPPEL study), we found that patients and citizens have more permissive attitudes towards euthanasia in patients with advanced dementia than physicians. The KOPPEL study was published earlier [4]. However, the qualitative interview data regarding euthanasia in advanced dementia were not presented. The reasons behind the differing views on this issue thus remained unclear. Therefore, we analyzed the interview data from the KOPPEL study, guided by the following research question: What are the opinions of physicians and members of the general public in the Netherlands on euthanasia in patients with advanced dementia? We analysed the opinions of the respondents with the aim to clarify the divergence between physicians and the general public.

**Table 1:** **Dutch criteria of due care for euthanasia and physician-assisted suicide (Termination of Life on Request and Assisted Suicide Act, 2002)**

Nr	Criterium of due care
**1**	The physician must be convinced that the patient’s request is voluntary and well-considered
**2**	The physician must be convinced that the patient’s suffering is unbearable and without prospect of relief
**3**	The patient must be informed about his/her situation and prospects
**4**	The physician and the patient together must be convinced that there is no reasonable alternative solution for the situation
**5**	At least one other independent physician must be consulted
**6**	The ending of life must be performed in a professionally careful way

## Methods

### Design and population

This study is part of the KOPPEL-study: Knowledge and Opinions of the Public and Professionals regarding End of Life decisions. The KOPPEL study is a mixed method study: quantitative methods were used to collect data on knowledge and opinions about euthanasia among the Dutch general public, physicians and nurses (see Additional files 1 and 2). To gain more in-depth information about the views of these groups, we performed qualitative interviews among selected respondents [4].

We used purposive sampling to select candidates [5] with the aim to maximize the range of different opinions and experiences. Furthermore, we strived for a balanced distribution of age, education and gender. We selected 125 respondents in total. We continued enrolling subjects for interviews in each group until conceptual saturation per group was achieved. Methods are described in more detail in a previous publication.

### Data collection

Four researchers (PK, NR, DvT, and HW) and two medical students conducted the interviews. The one-hour interviews were semi-structured with use of an interview guideline with open questions and topics (see Additional file 3). Most interviews with physicians were conducted at their working place and with members of the general public in their homes. A random sample of respondents was presented with the following vignette about a patient with advanced dementia with an AED containing a request for euthanasia that was eventually granted by his physician.

Mr Smit is 62 years old and suffering from dementia. He doesn’t recognise his wife and children anymore, refuses to eat and withdrawals into himself more and more. It is no longer possible to communicate with him about his treatment. Shortly before he became demented, he drafted an advance directive with a euthanasia request in case of dementia. His family agrees. The physician decides to honour his patient’s advance directive and performs euthanasia.

Respondents were asked two questions about the vignette: “Is the physician’s act legal in the Netherlands?” and “Do you personally agree with the physician’s act?” (see Additional file 4).

### Analysis

All interviews have been transcribed verbatim and were analysed with content analysis using Atlas.ti version 6.1.1. A uniform code tree was developed and agreed upon by all researchers. The interview parts including the vignettes were analysed in more detail by two researchers independently (PK and NR). Themes and coding were discussed until consensus was reached in all cases. The findings were later discussed with other members of the group (HD, AH, JR, GT).

### Ethics approval and informed consent

Regarding ethical approval, according to the Dutch Medical Research Involving Human Subjects Act, this kind of observational study is exempt from ethical review.

Respondents were selected from the participants of the KOPPEL study, who voluntarily provided us with their personal contact details. Before the start of the interview, the voluntary character and confidentiality of participation were emphasized. Study participants were informed about the aims, content, procedure and publication of the study. Participants provided oral informed consent to participation in the study. Interviews were recorded after obtaining the permission of the interviewee*.*

## Results

### Respondents’ characteristics

All interviewees were asked about their opinions about the vignette on euthanasia in a patient with advanced dementia. Saturation in the KOPPEL-interview study was reached after conducting 67 interviews: 16 with medical specialists, 19 with general practitioners, 16 with elderly care physicians and16 with members of the general public. In addition, 18 nurses were interviewed, but the results are not presented here. See Table 2 for interviewees’ characteristics.

**Table 2:** **Background characteristics of interview respondents**

	Physicians (n = 49) N (%)	Members of the general public (n = 16) (%) N
**Age**		
Mean ± SD	49 ± 9	54 ± 13
**Gender**		
Male	33 (67)	8 (50)
Female	16 (33)	8 (50)
**Education** ^**1**^		
Low	n.a.	5 (31)
Middle	n.a.	6 (38)
High	49 (100)	5 (31)
**Experience with euthanasia request** ^**3**^		
Yes	38 (78)	4 (25)
No	10 (20)	12 (75)
Unknown	1 (2)	n.a.
**Experience with advance directive** ^**4**^		
Yes	19 (30)	1 (6)
No	26 (53)	14 (88)
Unknown	4 (8)	1 (6)
**Care setting**		
Hospital care^**2**^	16 (33)	n.a.
Home care	19 (39)	n.a.
Nursing home care	14 (28)	n.a.
Unknown	n.a.	n.a.
**Attitude towards euthanasia and physician-assisted suicide**		
Liberal	23 (47)	8 (50)
Intermediate	0 (0)	3 (19)
Conservative	22 (45)	5 (31)
Unknown	4 (8)	n.a.

^**1**^Low = level 1-3 according to ISCED guidance (primary school, lower secondary general education, lower vocational education), middle = level 4 according to ISCED guidance (intermediate vocational or higher secondary general education), high = level 5-7 according to ISCED guidance (higher vocational education or university).

^**2**^internal medicine (2), sugery (1), neurology (5), pulmonology (5), cardiology (3).

^3^Experience with a patient’s (for physicians) or relative’s (for members of the general public) actual request in the last 5 years.

^4^Experience with an incompetent patient’s (for physicians) or relative’s (for members of the general public) advance directive in the last 5 years, in a situation where a medical decision needed to be made.

Many physicians emphasized that they consider euthanasia in advanced dementia problematic, both legally and personally. They doubted if the criteria of due care can be met in this situation. Specifically mentioned was the complicated assessment of whether the criteria concerning unbearable suffering without prospect of relief and the voluntariness of the request are met. Almost all physicians mentioned the need for explicit confirmation of the euthanasia request by the patient at the time of decision making as well as confirmation of the unbearableness of suffering. They explained that such communication is generally impossible in patients with advanced dementia. Some physicians emphasized the importance of high quality supportive care at the end of life and that euthanasia should not be viewed as a substitute for good care. Several physicians said that they feel that the general public is not well informed about the limitations of the law in case of late stage dementia. Some physicians stated that euthanasia in advanced dementia should be possible, even if they (incorrectly) thought this is not legally allowed in the Netherlands (see Table 3).

**Table 3:** **Euthanasia in advanced dementia: Examples of interviewee responses**

Topic	Interviewee response
Unbearable suffering	*If you see elderly people who have gone downhill and behave like small children, you say, “I don’t want that”. So then there has to be the option that if you become like that, you can say, “Just give me a pill or an injection or whatever”. (member of the public)*
	*I find it very difficult to determine whether a patient with dementia suffers unbearably. I tried to find that out in my father’s case, but I never got an idea if he, and all the patients around him of course, if they are suffering? (member of the public)*
	*I see people and think: I don’t think you are suffering, the family is suffering and others around him, because the person goes downhill, but at that moment I can not assess if the patient is still suffering that much and if it is really unbearable. (general practitioner)*
	*Is psychological suffering also unbearable suffering? Is someone who has dementia, but doesn’t know that about himself, is he suffering unbearably? (medical specialist)*
Voluntary and well-considered request	*Because in my view, one should be able to decide deliberately that one’s decision still stands. That it hasn’t changed. And an elderly person with dementia cannot do this. (elderly care physician)*
	*I always explain, if someone is suffering from dementia, an advance euthanasia directive does not apply. The person cannot ask him- or herself for euthanasia anymore. I cannot kill anyone who does not, who maybe doesn’t want that anymore now. (elderly care physician)*
Communication	*So it is not as much the directive but rather that you have to be in touch with the patient and have to have that conversation about whether you indeed consider your life to be unbearable. (elderly care physician)*
	*Look, such a euthanasia directive exists, but that request must of course be repeated at the moment itself, otherwise you could come up with such a directive at any time and say, well, now it has to end. (elderly care physician)*
Societal factors	*There are situations known where they still have to get the people out of bed at twelve for lunch, they have no time, well then they lie, for example, the whole night in a diaper full of shit. You don’t want that kind of life and that there is nothing you can do. Well then you feel embarrassed right? (member of the public)*
	*There are people who are just lonely and never have any visitors. But the moment you accept that those people then should get euthanasia, then you’re at the wrong end of the process. Instead, you have to make sure that it [loneliness] doesn’t occur anymore. (general practitioner)*
Ethical considerations	*Some tendency will develop in the Netherlands saying that the lives of people with Alzheimer’s disease living in a nursing home don’t count anymore and that a life like that is not meaningful anymore. And I’m against that. There is a noticable change of view on Alzheimer. And that is one of the reasons why I oppose to euthanasia in Alzheimer patients. Because a judgement will be made: this life is not meaningful anymore. (general practitioner)*
The physician’s role	*If someone asks me „If I become demented then you really have to give me an injection or whatever“, well, then I can say „I’m sorry, but I’m reluctant to do that. I was taught to cure you and not to let you die, but let’s agree that if you will be in such a condition and you have dementia and suffer from a serious airway infection, then I will not let you live any longer.“ (elderly care physician)*
The role of the law	*I think it is inconsistent, look, such an advance directive is legal, but the law also states that the physician has to be convinced of the hopelessness and unbearableness of the patient’s suffering. And if you can’t have a conversation about that, then you can’t get convinced and therefore can’t perform euthanasia. (elderly care physician)*

Several members of the general public described a negative image of advanced dementia: they mentioned suffering they had personally witnessed in friends or relatives with dementia and many of these interviewees called these situations humiliating. Some members of the general public mentioned the absence of a prospect of relief of suffering due to dementia as an argument in favor of euthanasia in case of advanced dementia. Other respondents mentioned that they thought the suffering was absent or doubtful in encounters with their friends or relatives with advanced dementia. Some respondents claimed that the request of a formerly competent person should be respected and that patients with advanced dementia should be able to get euthanasia if they so desire (see Table 3).

Both physicians and members of general public recognized the importance of respecting a competent wish as laid down in an AED. However, both also mentioned possible problems and limitations regarding a formerly written request: foreseeing future wishes and suffering was regarded as difficult, because people may change their preferences and adapt to new situations they previously thought to be unbearable. Furthermore, they questioned if suffering is unbearable in patients with advanced dementia or mentioned that especially the relatives suffer. Both groups perceived the quality of care in some nursing homes as inferior and pleaded for better care for patients with dementia. Other issues they mentioned were the unfinished societal debate about euthanasia and the problem of the growing prevalence of dementia. Respondents of both groups said they would prefer using the AED as a non-treatment directive instead of an euthanasia directive. They also stated that performing euthanasia in a patient with advanced dementia would be burdensome for the physician.

## Discussion

Earlier research showed a majority of members of the general public personally agree with euthanasia on the basis of an AED in case of advanced dementia, whereas only a minority of physicians does [4, 6, 7]. In our interviews, both physicians and members of the general public acknowledge difficulties in the assessment of the voluntariness of the request and the extent of suffering of patients with advanced dementia. Physicians regard direct communication with the patient as essential for this assessment. Obviously, this is compromised in patients with advanced dementia.

No less than 67 interviews were conducted and the method of purposive sampling guaranteed a wide range of opinions. Questions were highly comparable between all groups of respondents, ensuring the validity of the comparison. Furthermore, the use of vignettes in decision-making research has shown its value [8].

Respondents were not fully representative of the Dutch population; the sample was slightly older, more often male, higher educated and more often shared a household. Migrants also were underrepresented. For all groups, possible selection bias should be taken into account. It could be that people with more experience and affinity with the discussion about euthanasia were more likely to participate in this study.

Previous research hypothesized that communication with the patient is important for physicians and that euthanasia therefore will be only rarely performed in patients with advanced dementia [1, 9–11]. Our in-depth interview study confirms this hypothesis. The criteria of due care regarding voluntariness and unbearable suffering make communication of key importance for doctors in the decision-making process. Unbearable suffering and a voluntary euthanasia request of a patient are apparently criteria that should be jointly fulfilled for physicians to experience a moral appeal that is strong enough to be willing to perform euthanasia. By communicating personally with the patient in one or more conversations, the physician acquires a comprehensive understanding of the patient’s suffering and his or her wish to die. This understanding then moves the physician to become willing to perform euthanasia. An AED, however, contains a request that was expressed in the past when the patient was still competent, and provides no information about the patient’s actual suffering. In a patient with advanced dementia, the physician thus needs to be moved literally and figuratively by his own perception of the patients’ suffering combined with what is stated in the AED. Both elements that construct a moral appeal on the physician are present in a rather indirect way only. According to the interviews in this study, respondents from both groups acknowledge the difficulty of assessing whether there is unbearable suffering in advanced dementia. De Boer et al. found that 54% of elderly care physicians agreed with the statement ‘It is impossible to determine whether an incompetent person experiences his/her dementia as unbearable and hopeless suffering’ and 76% agreed with the statement ‘It is impossible to determine at what moment an AED in dementia is to be carried out’ [9]. Livingston et al. underpinned this and found that people with Alzheimer’s disease actually tend to rate their quality of life high, even though most outsiders would classify their daily existence as undesirable [12]. At the same time it is fair to say that end-stage dementia patients probably have a less than optimal quality of life, in part because of suboptimal care [12].

Furthermore, physicians as well as members of the general public acknowledge the limits of AEDs: at the time of writing such a document one does not know the future nor to what extent one will be able to adjust to new situations. Previous research shows that even patients with dementia adjust actively to their disease, using both emotion- and problem-oriented strategies [13]. The previously anticipated experiences of patients with advanced dementia may thus differ from their actual daily experiences, but the physician is generally not able to discuss these potential changes with the patient.

In some cases suffering may be unambiguously present and understood by non-verbal communication. However, the assessment of the *unbearableness* of that suffering, which is a prerequisite for euthanasia, seems to remain an important challenge.

Without adequate conversation about the patient’s wishes and their experiences of suffering, making decisions about another person’s death apparently goes beyond what physicians think they can account for.

All respondents seem to be guided by the best interest of the patient. However, different roles and responsibilities in the decision-making process and performance of euthanasia are likely to play a role, as has been suggested before [14]. Performing euthanasia is known to have a clear emotional impact on physicians [13] and this may at least partly explain their reticence. When the patient is no longer capable of confirming his wish, the burden may be weightier. Furthermore, fear of legal consequences, due to the experienced difficulties in meeting the criteria of due care, may hold physicians back in performing euthanasia in cases of late-dementia.

Earlier research showed a clear discrepancy between the general public and physicians in their support for euthanasia in a patient with advanced dementia. However, our interview study showed many similarities in terms of appreciating the difficulties in assessing voluntariness and unbearable suffering.

## Conclusion

Legally, an AED could replace direct communication with the patient when making decisions about euthanasia. In practice, adequate verbal communication with the patient appears to be essential for physicians. Performing euthanasia in a case where the presence of unbearable suffering and voluntariness of the request cannot be directly confirmed by the patient is a bridge too far for most of them. For this reason, the applicability of AEDs in advanced dementia seems limited, which explains the very low number of cases. Physicians and members of the general public acknowledge the same difficulties but may have different expectations about the possibility of euthanasia in late stage dementia. Respectful communication between all involved remains important for a better understanding of the (im) possibilities of ending life in advanced dementia and for the prevention of expectations that cannot be met.

## Electronic supplementary material

Additional file 1: **Questionnaire for physicians.** (PDF 142 KB)

Additional file 2: **Questionnaire for the general public.** (PDF 57 KB)

Additional file 3: **Interview guide.** (PDF 47 KB)

Additional file 4: **Translated vignette and questions.** (DOCX 14 KB)
