# Supplementary material for: Opinions about euthanasia and advanced dementia: a qualitative study among Dutch physicians and members of the general public
Source: BMC Med Ethics. 2015 Jan 28;16:7. doi: 10.1186/1472-6939-16-7 (PMC4350907; doi:10.1186/1472-6939-16-7)
Supplement: Supplementary file 1 — Additional file 1: Questionnaire for physicians. (PDF 142 KB) [file 12910_2014_328_MOESM1_ESM.pdf]

Universitair Medisch Centrum
Utrecht

Vragenlijst

KOPPEL-studie:
Kennis en Opvattingen van Publiek en Professionals
over Einde Leven beslissingen

Erasmus MC
Universitair Medisch Centrum Rotterdam

umcg

rijksuniversiteit
groningen

Inleiding op de vragenlijst

Deze vragenlijst is onderdeel van een onderzoek naar medische beslissingen rond het levenseinde. Dit onderzoek wordt in opdracht van het ministerie van VWS uitgevoerd door de universiteiten van Utrecht, Rotterdam en Groningen. Doel is na te gaan wat de kennis, opvattingen en ervaringen zijn van publiek, artsen, verpleegkundigen en verzorgenden. De uitkomsten van het onderzoek zullen worden meegenomen in het verdere beleid op het gebied van medische beslissingen rond het levenseinde in Nederland.

Uw deelname is van groot belang voor het slagen van dit onderzoek.

Bij de verwerking van deze vragenlijst blijft u volledig anoniem. Wij vragen u eerst naar uw eigen ervaringen. Vervolgens worden een aantal stellingen en een aantal korte casus aan u voorgelegd. Wij verwachten dat u hier ongeveer 25 minuten mee bezig zult zijn.

Bij voorbaat hartelijk dank voor uw tijd en aandacht!

Eigen ervaringen

1. Hebt u de afgelopen 5 jaar de medische zorg gehad voor één of meer patiënten in de laatste levensfase*?
   - ❑ Ja
   - ❑ Nee

   **Met laatste levensfase bedoelen we hier ongeveer het laatste half jaar van het leven.*

Euthanasie en hulp bij zelfdoding

2. Is de afgelopen 5 jaar door één of meer patiënten een verzoek tot euthanasie of hulp bij zelfdoding op afzienbare termijn (= actueel verzoek) aan u gericht?
   - ❑ Ja, 1 of 2 keer
   - ❑ Ja, 3 tot 5 keer
   - ❑ Ja, 6 tot 10 keer
   - ❑ Ja, meer dan 10 keer
   - ❑ Nee -> *ga naar vraag 15.*

3. Neem de *laatste* patiënt in gedachten die aan u een actueel verzoek tot euthanasie of hulp bij zelfdoding heeft gericht. Wat was bij benadering de leeftijd van deze patiënt?
   - ❑ 0-64 jaar
   - ❑ 65-79 jaar
   - ❑ 80 jaar of ouder

4. Wat was de diagnose van deze patiënt?
   (meer dan één antwoord toegestaan)
   - ❑ Kanker
   - ❑ Lichamelijke aandoening, maar geen kanker
   - ❑ Dementie
   - ❑ Psychiatrische aandoening, maar geen dementie
   - ❑ Geen medisch classificeerbare aandoening
   - ❑ Anders, namelijk ……………………………………

5. Wat was de *belangrijkste* oorzaak van het lijden van de patiënt?
(één antwoord kiezen)
- ❑ Lichamelijk klacht zoals pijn of benauwdheid
- ❑ Angst voor toekomstig lijden
- ❑ Zorgafhankelijkheid
- ❑ Last voor anderen
- ❑ Verlies van waardigheid
- ❑ Angst de regie te verliezen
- ❑ Uitzichtloosheid van de situatie
- ❑ Levensmoeheid
- ❑ Anders, namelijk ……………………

6. Welke van onderstaande (positieve) ervaringen hebt u tijdens het besluitvormingsproces* gehad?
**rond het verzoek om euthanasie of hulp bij zelfdoding*

		Ja	Nee/ n.v.t.
a)	Ik heb een goede vertrouwensrelatie op kunnen bouwen met de patiënt	❑	❑
b)	Er was voldoende tijd om tot een weloverwogen besluit te komen	❑	❑
c)	Er was respect van de patiënt voor mijn positie als arts in dit proces	❑	❑
d)	Er was respect van de naasten van de patiënt voor mijn positie als arts in dit proces	❑	❑
e)	Er was overeenstemming over het uiteindelijke besluit met verpleging en/of verzorging	❑	❑
f)	Er was overeenstemming over het uiteindelijke besluit met de naasten van de patiënt	❑	❑
g)	Ik voelde mij gesteund door mijn instelling/ samenwerkingsverband in het besluitvormingsproces	❑	❑
h)	Andere positieve ervaring(en), namelijk …………………………………		

7. Welke van onderstaande (negatieve) ervaringen hebt u tijdens het besluitvormingsproces* gehad?
**rond het verzoek om euthanasie of hulp bij zelfdoding*

		Ja	Nee/ n.v.t.
a)	Ik heb onvoldoende een vertrouwensrelatie op kunnen bouwen met de patiënt	❑	❑
b)	Er was onvoldoende tijd om tot een weloverwogen besluit te komen	❑	❑
c)	Ik had gewetensbezwaren	❑	❑
d)	Ik voelde me onder druk gezet door de patiënt	❑	❑
e)	Ik voelde me onder druk gezet door naasten van de patiënt	❑	❑
f)	Er was verschil van mening over het uiteindelijke besluit met verpleging en/of verzorging	❑	❑
g)	Er was verschil van mening over het uiteindelijke besluit met naasten van de patiënt	❑	❑
h)	Ik voelde mij gehinderd door mijn instelling/ samenwerkingsverband	❑	❑
i)	Andere negatieve ervaring(en), namelijk …………………………………		

8. Als u terugkijkt op het besluitvormingsproces, bent u dan tevreden?
   - ❑ Ja, heel tevreden
   - ❑ Ja, tevreden
   - ❑ Niet tevreden en niet ontevreden
   - ❑ Nee, ontevreden
   - ❑ Nee, heel ontevreden

9. Hebt u *bij deze patiënt* euthanasie of hulp bij zelfdoding uitgevoerd?
   - ❑ Ja, euthanasie -> *ga naar vraag 11.*
   - ❑ Ja, hulp bij zelfdoding -> *ga naar vraag 14.*
   - ❑ Nee -> *ga naar vraag 10.*

10. Wat is/ zijn de reden(en) dat u het verzoek om euthanasie of hulp bij zelfdoding niet hebt uitgevoerd?

		Ja	Nee/ n.v.t.
a)	De patiënt overleed op natuurlijke wijze voordat euthanasie of hulp bij zelfdoding kon worden uitgevoerd	❑	❑
b)	Ik was onvoldoende overtuigd van de vrijwilligheid of weloverwogenheid van het verzoek	❑	❑
c)	De patiënt trok het verzoek in	❑	❑
d)	Ik was onvoldoende overtuigd van de ondraaglijkheid van het lijden	❑	❑
e)	Ik was onvoldoende overtuigd van de uitzichtloosheid van het lijden	❑	❑
f)	Ik zag nog een redelijke andere oplossing	❑	❑
g)	Volgens de consulent was onvoldoende aan de zorgvuldigheidseisen voldaan	❑	❑
h)	Naasten van de patiënt waren het oneens met uitvoering van het verzoek om euthanasie of hulp bij zelfdoding	❑	❑
i)	Ik zag teveel op tegen het uitvoeren van euthanasie of hulp bij zelfdoding	❑	❑
j)	Ik zag teveel op tegen de meldingsprocedure voor euthanasie of hulp bij zelfdoding	❑	❑
k)	Ik had gewetensbezwaren	❑	❑
l)	Mijn instelling/ samenwerkingsverband is tegen euthanasie of hulp bij zelfdoding	❑	❑
m)	Andere situatie, namelijk …………………………………………………..		

-> *Ga naar vraag 14.*

11. Hebt u de mogelijkheid van hulp bij zelfdoding in plaats van euthanasie aan de patiënt voorgesteld?
   - ❑ Ja
   - ❑ Nee -> *ga naar vraag 13.*

12. Waarom is voor euthanasie en niet voor hulp bij zelfdoding gekozen?

-> *ga naar vraag 14.*

13. Waarom is de mogelijkheid van hulp bij zelfdoding niet aan de patiënt voorgesteld?

14. Hoe beoordeelt u achteraf de kwaliteit van sterven van deze patiënt?
   - ❑ Heel goed
   - ❑ Goed
   - ❑ Niet goed en niet slecht
   - ❑ Slecht
   - ❑ Heel slecht

   - ❑ Weet ik niet/ n.v.t.

Palliatieve sedatie

15. Hebt u de afgelopen 5 jaar bij één of meer patiënten continue diepe sedatie tot aan het overlijden overwogen?
   - ❑ Ja
   - ❑ Nee -> *ga naar vraag 25.*

16. Neem de *laatste* patiënt in gedachten bij wie u continue diepe sedatie tot aan het overlijden hebt overwogen. Wat was bij benadering de leeftijd van de patiënt?
   - ❑ 0-64 jaar
   - ❑ 65-79 jaar
   - ❑ 80 jaar of ouder

17. Wat was de aard van het lijden waarvoor deze vorm van sedatie *vooral* werd overwogen? *(één antwoord kiezen)*
   - ❑ Lichamelijk lijden
   - ❑ Psychisch lijden
   - ❑ Existentieel lijden*
   - ❑ Anders, namelijk …………………..

   **Lijden aan gebrek aan zingeving*

18. Welke van onderstaande (positieve) ervaringen hebt u tijdens het besluitvormingsproces* gehad?
rond de overweging van continue diepe sedatie tot aan het overlijden

		Ja	Nee/ n.v.t.
a)	Er was voldoende tijd om tot een weloverwogen besluit te komen	❑	❑
b)	Er was overeenstemming over het uiteindelijke besluit met verpleging en/of verzorging	❑	❑
c)	Er was overeenstemming over het uiteindelijke besluit met de naasten van de patiënt	❑	❑
d)	Ik voelde mij gesteund door mijn instelling/ samenwerkingsverband in het besluitvormingsproces	❑	❑
e)	Andere positieve ervaring(en), namelijk		

19. Welke van onderstaande (negatieve) ervaringen hebt u tijdens het besluitvormingsproces* gehad?
rond de overweging van continue diepe sedatie tot aan het overlijden

		Ja	Nee/ n.v.t.
a)	Er was onvoldoende tijd om tot een weloverwogen besluit te komen	❑	❑
b)	Ik had gewetensbezwaren	❑	❑
c)	Ik voelde me onder druk gezet door de patiënt	❑	❑
d)	Ik voelde me onder druk gezet door naasten van de patiënt	❑	❑
e)	Er was verschil van mening over het uiteindelijke besluit met verpleging en/of verzorging	❑	❑
f)	Er was verschil van mening over het uiteindelijke besluit met naasten van de patiënt	❑	❑
g)	De patiënt wilde liever euthanasie of hulp bij zelfdoding en kwam hier ook voor in aanmerking	❑	❑
h)	De patiënt wilde liever euthanasie of hulp bij zelfdoding, maar kwam hier niet voor in aanmerking	❑	❑
i)	Andere negatieve ervaring(en), namelijk		

20. Als u terugkijkt op het besluitvormingsproces, bent u dan tevreden?
❑ Ja, heel tevreden
❑ Ja, tevreden
❑ Niet tevreden en niet ontevreden
❑ Nee, ontevreden
❑ Nee, heel ontevreden

21. Hebt u *bij deze patiënt* continue diepe sedatie tot aan het overlijden uitgevoerd?
❑ Ja -> *ga naar vraag 23.*
❑ Nee

22. Wat is/ zijn de reden(en) dat u de continue diepe sedatie tot aan het overlijden niet hebt uitgevoerd?

		Ja	Nee
a)	De patiënt overleed op natuurlijke wijze voordat de sedatie kon worden uitgevoerd	❑	❑
b)	De symptomen waren op dat moment naar mijn mening onvoldoende aan te merken als refractair	❑	❑
c)	Ik had moeite met het inschatten van de levensverwachting	❑	❑
d)	Ik zag teveel op tegen het uitvoeren van de sedatie	❑	❑
e)	De patiënt was het er mee oneens dat het bewustzijn verlaagd zou worden	❑	❑
f)	De naasten waren het er mee oneens	❑	❑
g)	Er is uiteindelijk voor euthanasie gekozen	❑	❑
h)	Ik had gewetensbezwaren	❑	❑
i)	Andere situatie, namelijk …………………………………………………………………		

-> *Ga naar vraag 24.*

23. Hebt u daarbij kunstmatig vocht en voeding laten toedienen?
❑ Ja
❑ Nee

24. Hoe beoordeelt u achteraf de kwaliteit van sterven van deze patiënt?
❑ Heel goed
❑ Goed
❑ Niet goed en niet slecht
❑ Slecht
❑ Heel slecht

❑ Weet ik niet/ n.v.t.

Wilsverklaringen

25. Is er in de afgelopen 5 jaar door één of meer patiënten een schriftelijke wilsverklaring aan u voorgelegd over een situatie die *mogelijk in de toekomst* aan de orde zou kunnen komen?
(meer dan één antwoord toegestaan)
❑ Ja, schriftelijke behandelweigering: niet-reanimeren
❑ Ja, schriftelijke behandelweigering anders dan niet-reanimeren
❑ Ja, schriftelijke euthanasieverklaring
❑ Ja, schriftelijke levenswensverklaring (waarin levensbeëindiging als optie om lijden te verlichten wordt uitgesloten)
❑ Ja, schriftelijke aanwijzing van een vertegenwoordiger
❑ Andere wilsverklaring, namelijk ……………………………..
❑ Nee -> *ga naar vraag 27.*

26. Neem de *laatste* patiënt in gedachten die een wilsverklaring aan u heeft voorgelegd over een situatie die *mogelijk in de toekomst* aan de orde zou kunnen komen. Welke van de volgende dingen hebt u toen gedaan?

		Ja	Nee/ n.v.t.
a)	Ik heb hierover een gesprek gehad met de patiënt	❑	❑
b)	Ik heb de wilsverklaring opgeborgen in het archief	❑	❑
c)	Ik heb de patiënt geadviseerd ook naasten te informeren	❑	❑
d)	Ik heb de patiënt geadviseerd ook andere behandelend artsen te informeren	❑	❑
e)	Ik heb de patiënt geadviseerd gebruik te maken van hulpmiddelen om het bestaan van de wilsverklaring in acute situaties snel kenbaar te maken (zoals een pasje in de portemonnee)	❑	❑
f)	Ik heb de patiënt geadviseerd de wilsverklaring regelmatig te actualiseren	❑	❑
g)	Ik heb bij (terug)verwijzing zelf de betreffende arts/ instelling geïnformeerd	❑	❑
h)	Anders, namelijk …………………………………………………………………………………………		

De volgende vragen gaan over de situatie van een *wilsonbekwame* patiënt.

27. Hebt u de afgelopen 5 jaar een situatie meegemaakt waarin een medische beslissing genomen moest worden bij een wilsonbekwame patiënt die in het bezit was van een schriftelijke wilsverklaring?

❑ Ja, 1 of 2 keer
❑ Ja, 3 tot 5 keer
❑ Ja, 6 tot 10 keer
❑ Ja, meer dan 10 keer
❑ Nee -> *ga naar vraag 32.*

28. Neem de *laatste* wilsonbekwame patiënt in gedachten die in het bezit was van een schriftelijke wilsverklaring en bij wie een medische beslissing genomen moest worden. Wat voor wilsverklaring was dat?
(meer dan één antwoord toegestaan)

❑ Schriftelijke behandelweigering: niet-reanimeren
❑ Schriftelijke behandelweigering anders dan niet-reanimeren
❑ Schriftelijke euthanasieverklaring
❑ Schriftelijke levenswensverklaring (waarin levensbeëindiging als optie om lijden te verlichten wordt uitgesloten)
❑ Schriftelijke aanwijzing van een vertegenwoordiger
❑ Andere wilsverklaring, namelijk ....

Indien u slechts één antwoord heeft aangekruist -> ga naar vraag 29.

28a. Welke wilsverklaring was het meest relevant voor de medische beslissing die genomen moest worden?
(één antwoord kiezen)

❑ Schriftelijke behandelweigering: niet-reanimeren
❑ Schriftelijke behandelweigering anders dan niet-reanimeren
❑ Schriftelijke euthanasieverklaring
❑ Schriftelijke levenswensverklaring (waarin levensbeëindiging als optie om lijden te verlichten wordt uitgesloten)

❑ Schriftelijke aanwijzing van een vertegenwoordiger
❑ Andere wilsverklaring

29. Hebt u bij de besluitvorming *bij deze patiënt* de volgende ervaringen gehad of constateringen gedaan (met betrekking tot de (meest relevante) wilsverklaring)?

		Ja	Nee/ n.v.t.
a)	Het was duidelijk dat het document werkelijk van de betreffende patiënt afkomstig was	❑	❑
b)	Het was duidelijk dat de patiënt ten tijde van het opstellen van het document wilsbekwaam was	❑	❑
c)	Het was duidelijk dat het document van toepassing was in deze situatie	❑	❑
d)	Het was duidelijk wat volgens het document de (on)gewenste handeling was in deze situatie	❑	❑
e)	Het document was (mogelijk) te oud, namelijk ongeveer …… jaar oud	❑	❑
f)	De wilsverklaring was inhoudelijk strijdig met mijn medische oordeel als arts	❑	❑
g)	De wilsverklaring was strijdig met de wens van naasten van de patiënt	❑	❑
h)	Andere ervaringen, namelijk……………………………………………………………….		

30. Hebt u in deze situatie de wilsverklaring uiteindelijk gevolgd?
❑ Ja
❑ Nee
❑ Anders, namelijk …

31. Zou u een andere medische beslissing genomen hebben als er *geen* wilsverklaring was geweest?
❑ Ja, zeker wel
❑ Waarschijnlijk wel
❑ Weet ik niet
❑ Waarschijnlijk niet
❑ Nee, zeker niet

Kennis en opvattingen

Met de volgende vragen willen wij graag uw kennis en opvattingen toetsen. De kennisvragen vormen geen examen, dus zoekt u niets op en vult u alstublieft in wat u uit uw hoofd weet of denkt te weten.

Palliatieve zorg

Volgens de WHO-definitie palliatieve zorg...

	Juist	Onjuist	Weet ik niet
32. ...is het doel van palliatieve zorg alleen pijnbestrijding.	❑	❑	❑
33. ...vindt palliatieve zorg alleen plaats rond het levenseinde.	❑	❑	❑
34. ...omvat palliatieve zorg ook ondersteuning bij problemen van spirituele aard.	❑	❑	❑
35. ...omvat palliatieve zorg ook ondersteuning van de naasten van de patiënt.	❑	❑	❑

Naar mijn mening …	Helemaal oneens	Oneens	Noch eens, noch oneens	Eens	Helemaal eens	Weet ik niet
36. ...is het doel van palliatieve zorg alleen pijnbestrijding.	❑	❑	❑	❑	❑	❑
37. ...vindt palliatieve zorg alleen plaats rond het levenseinde.	❑	❑	❑	❑	❑	❑
38. ...omvat palliatieve zorg ook ondersteuning bij problemen van spirituele aard.	❑	❑	❑	❑	❑	❑
39. ...omvat palliatieve zorg ook ondersteuning van de naasten van de patiënt.	❑	❑	❑	❑	❑	❑
40. ...is kunstmatige toediening van vocht en voeding basiszorg en geen medische behandeling.	❑	❑	❑	❑	❑	❑

Euthanasie en hulp bij zelfdoding

Volgens de Nederlandse euthanasiewet en jurisprudentie (rechterlijke uitspraken) …	Juist	Onjuist	Weet ik niet
41. ...heeft iedereen die dat wil recht op euthanasie of hulp bij zelfdoding.	❑	❑	❑
42. ...mag euthanasie of hulp bij zelfdoding alleen worden uitgevoerd bij een patiënt die nog maar enkele weken te leven heeft.	❑	❑	❑
43. ...is euthanasie of hulp bij zelfdoding alleen toegestaan als een patiënt daar zelf om vraagt.	❑	❑	❑
44. …mag de verpleegkundige in opdracht van de arts de dodelijke middelen toedienen bij euthanasie.	❑	❑	❑
45. …is een arts die principieel weigert euthanasie of hulp bij zelfdoding uit te voeren, verplicht de patiënt door te verwijzen naar een andere arts.	❑	❑	❑

Naar mijn mening …	Helemaal oneens	Oneens	Noch eens, noch oneens	Eens	Helemaal eens	Weet ik niet
46. …heeft ieder mens het recht om zelf te beschikken over eigen leven en dood.	❑	❑	❑	❑	❑	❑
47. …moet iedereen die dat wil het recht hebben op euthanasie of hulp bij zelfdoding.	❑	❑	❑	❑	❑	❑
48. …mag euthanasie of hulp bij zelfdoding alleen worden uitgevoerd bij een patiënt die nog maar enkele weken te leven heeft.	❑	❑	❑	❑	❑	❑
49. …is euthanasie of hulp bij zelfdoding alleen toegestaan als een patiënt daar zelf om vraagt.	❑	❑	❑	❑	❑	❑
50. …moet het toegestaan zijn dat de verpleegkundige in opdracht van de arts de dodelijke middelen toedient bij euthanasie.	❑	❑	❑	❑	❑	❑
51. …moet het initiatief om euthanasie of hulp bij zelfdoding te bespreken altijd van de patiënt zelf uit gaan.	❑	❑	❑	❑	❑	❑
52. …moeten artsen hun patiënten in een vroeg stadium informeren over hun principiële bereidheid of weigering om euthanasie of hulp bij zelfdoding uit te voeren.	❑	❑	❑	❑	❑	❑
53. …is een arts die principieel weigert euthanasie of hulp bij zelfdoding uit te voeren, verplicht de patiënt door te verwijzen naar een andere arts.	❑	❑	❑	❑	❑	❑
54. ...heeft *in principe* hulp bij zelfdoding de voorkeur boven euthanasie.	❑	❑	❑	❑	❑	❑

55. Welke van de volgende omschrijvingen is het meest op u van toepassing?
(één antwoord kiezen)

❑ Ik beschouw mezelf als liberaal als het gaat om euthanasie en hulp bij zelfdoding
❑ Ik ben terughoudend als het gaat om euthanasie en hulp bij zelfdoding
❑ Ik ben een principiële tegenstander van euthanasie en hulp bij zelfdoding

56. Welke van de volgende omschrijvingen is het meest op u van toepassing?
(één antwoord kiezen)
- ❑ Ik heb wel eens euthanasie of hulp bij zelfdoding uitgevoerd
- ❑ Ik acht het denkbaar dat ik ooit euthanasie of hulp bij zelfdoding zou kunnen uitvoeren.
- ❑ Ik acht het *on*denkbaar dat ik ooit euthanasie of hulp bij zelfdoding zou kunnen uitvoeren.

57. Informeert u uw patiënten over uw principiële bereidheid of weigering om euthanasie of hulp bij zelfdoding uit te voeren?

		Ja	Nee
a)	Ja, in een kennismakingsgesprek of in de folder van de instelling of praktijk	❑	❑
b)	Ja, wanneer de patiënt daar zelf om vraagt	❑	❑
c)	Ja, wanneer de patiënt een verzoek aan mij richt om euthanasie of hulp bij zelfdoding voor een moment dat mogelijk in de toekomst aan de orde zou kunnen komen	❑	❑
d)	Ja, wanneer de patiënt een verzoek aan mij richt om euthanasie of hulp bij zelfdoding op afzienbare termijn	❑	❑
e)	Ja, anders, namelijk	❑	❑

f) Nee, ik spreek dit niet duidelijk uit ❑

58. Als aan de zorgvuldigheidseisen is voldaan en het medisch-technisch mogelijk is, heeft u dan een voorkeur voor één van de volgende handelwijzen?
- ❑ De arts geeft de patiënt een dodelijke injectie
- ❑ De patiënt drinkt het dodelijke drankje zelf op
- ❑ Geen voorkeur
- ❑ Anders, namelijk ....

59. Welke van onderstaande stellingen *zouden voor u een reden kunnen zijn* om voor hulp bij zelfdoding te kiezen in plaats van voor euthanasie?

		Ja	Nee	Weet ik niet
a)	Ik vind dat hulp bij zelfdoding de autonomie van de patiënt onderstreept	❑	❑	❑
b)	Ik vind dat hulp bij zelfdoding de vrijwillige keuze van de patiënt onderstreept	❑	❑	❑
c)	Ik vind dat hulp bij zelfdoding de eigen verantwoordelijkheid van de patiënt onderstreept	❑	❑	❑
d)	Ik beschouw hulp bij zelfdoding psychologisch gezien als minder belastend voor de arts	❑	❑	❑
e)	Ik beschouw hulp bij zelfdoding juridisch gezien als minder belastend voor de arts	❑	❑	❑
f)	Ik beschouw hulp bij zelfdoding wat de uitvoering betreft als minder belastend voor de arts	❑	❑	❑
g)	Hulp bij zelfdoding kan zorgen voor een minder abrupt stervensproces	❑	❑	❑
h)	Anders, namelijk …………………………………………………………………………………			

60. Welke van onderstaande stellingen *zouden voor u een reden kunnen zijn* om voor euthanasie te kiezen in plaats van voor hulp bij zelfdoding?

		Ja	Nee	Weet ik niet
a)	Ik vind het dodelijke middel bij hulp bij zelfdoding patiëntonvriendelijk	❑	❑	❑
b)	Ik vind het dodelijke middel bij hulp bij zelfdoding onbetrouwbaar	❑	❑	❑
c)	Bij hulp bij zelfdoding kan het lang duren voordat de dood intreedt (voor de patiënt)	❑	❑	❑
d)	Bij hulp bij zelfdoding kan het lang duren voordat de dood intreedt (voor de arts)	❑	❑	❑
e)	Veel patiënten *kunnen* volgens mij het dodelijke middel niet zelf tot zich nemen	❑	❑	❑
f)	Veel patiënten *willen* volgens mij het dodelijke middel niet zelf tot zich nemen	❑	❑	❑
g)	Anders, namelijk …………………………………………………………………………………………			

61. Wat verstaat u onder de term hulp bij zelfdoding?

		Ja	Nee
a)	Informatie geven over bronnen (bijvoorbeeld websites) waar men informatie kan vinden over methoden om een einde aan het leven te maken	❑	❑
b)	Uitleg geven over de mogelijkheden en het proces van versterven (stoppen met eten en drinken)	❑	❑
c)	Een advies geven over dodelijke middelen	❑	❑
d)	Een advies geven over andere methoden om een einde aan het leven te maken	❑	❑
e)	Een recept uitschrijven voor een dodelijk middel	❑	❑
f)	De patiënt een dodelijk drankje laten drinken, door mij aangereikt en onder mijn toezicht	❑	❑
g)	Anders, namelijk		

Palliatieve sedatie

De volgende vragen gaan over de richtlijn palliatieve sedatie van de KNMG (2009).

<u>Volgens de KNMG-richtlijn</u> palliatieve sedatie ...

	Juist	Onjuist	Weet ik niet
62. ...moet bij continue diepe sedatie tot aan het overlijden kunstmatig vocht en voeding worden toegediend om bespoediging van het overlijden te voorkomen.	❑	❑	❑
63. ...mag de verpleegkundige in opdracht van de arts de sederende middelen toedienen bij continue diepe sedatie tot aan het overlijden.	❑	❑	❑
64. ...spelen alleen onbehandelbare *lichamelijke* symptomen een rol in de indicatiestelling voor continue diepe sedatie tot aan het overlijden.	❑	❑	❑

Naar mijn mening …	Helemaal oneens	Oneens	Noch eens, noch oneens	Eens	Helemaal eens	Weet ik niet
65. ...moet bij continue diepe sedatie tot aan het overlijden kunstmatig vocht en voeding worden toegediend om bespoediging van het overlijden te voorkomen.	❑	❑	❑	❑	❑	❑
66. ...moet het toegestaan zijn dat de verpleegkundige in opdracht van de arts de sederende middelen toedient bij continue diepe sedatie tot aan het overlijden.	❑	❑	❑	❑	❑	❑
67. ...moeten alleen onbehandelbare *lichamelijke* symptomen een rol spelen in de indicatiestelling voor continue diepe sedatie tot aan het overlijden.	❑	❑	❑	❑	❑	❑
68. ...heeft *in principe* continue diepe sedatie tot aan het overlijden de voorkeur boven euthanasie of hulp bij zelfdoding (als de patiënt voor beide in aanmerking komt).	❑	❑	❑	❑	❑	❑

Wilsverklaringen

Het is mogelijk om in een schriftelijke wilsverklaring wensen vast te leggen over de medische zorg en behandeling in de laatste levensfase. Dit is vastgelegd in de WGBO.

Volgens de Wet op de Geneeskundige Behandelingsovereenkomst (WGBO)...	Juist	Onjuist	Weet ik niet
69. ...is een schriftelijke behandelweigering meer een verzoek aan de arts dan een dwingend voorschrift.	❑	❑	❑

Naar mijn mening …	Helemaal oneens	Oneens	Noch eens, noch oneens	Eens	Helemaal eens	Weet ik niet
70. ...is een schriftelijke behandelweigering meer een verzoek aan de arts dan een dwingend voorschrift.	❑	❑	❑	❑	❑	❑
71. ...moet elke schriftelijke wilsverklaring een vastgesteld model volgen.	❑	❑	❑	❑	❑	❑
72. ...moeten artsen meer initiatief nemen om patiënten op de mogelijkheid van het opstellen van een schriftelijke wilsverklaring te wijzen.	❑	❑	❑	❑	❑	❑

73. …moeten artsen meer initiatief nemen om een behandelweigering als niet-reanimeren of niet-beademen vast te leggen.	❑	❑	❑	❑	❑	❑

Algemeen

<u>Naar mijn mening</u> …	Helemaal oneens	Oneens	Noch eens, noch oneens	Eens	Helemaal eens	Weet ik niet
74. ...kan een arts beter beoordelen wat voor zijn/haar patiënt het beste is, dan dat de patiënt dit zelf van te voren kan bedenken.	❑	❑	❑	❑	❑	❑
75. ...mag een arts informatie voor de patiënt achterhouden, als hij denkt dat dit beter is voor de patiënt.	❑	❑	❑	❑	❑	❑

76. In de laatste levensfase worden vaak ingrijpende medische beslissingen genomen. Er kan verschillend gedacht worden over de manier waarop deze beslissingen tot stand zouden moeten komen.
Wilt u aangeven welke beschrijving van medische besluitvorming in de laatste levensfase u het meeste aanspreekt?
(één antwoord kiezen)
Ik vind dat ik als arts de patiënt goed moet informeren en dat…
❑ … ik als arts de beslissing moet nemen, op basis van wat ik denk dat het beste voor de patiënt is.
❑ … wij samen de beslissing moeten nemen, als een gezamenlijk besluit.
❑ … de patiënt zelf de beslissing moet nemen, op basis van wat hij denkt dat het beste voor hem is.

Casus

Tot slot volgen nog zes korte casus met vragen. Dit zijn verzonnen situaties, die in werkelijkheid kunnen voorkomen. U vindt deze casus op het inlegvel.

Persoonskenmerken

We besluiten met enkele vragen over uw persoonskenmerken.

95. Geslacht: ❑ man ❑ vrouw

96. Leeftijd: ….. jaar

97. Specialisme: …………………………….

98. Aantal jaren beroepservaring in huidig specialisme: …………..

99. Aantal werkuren per week werkzaam in dit specialisme: …………..

100. Bent u SCEN-arts? ❑ Ja ❑ Nee

Tot slot

Hartelijk dank voor het invullen van deze vragenlijst!

Wij zouden een aantal artsen die deze vragenlijst hebben ingevuld graag willen interviewen om de onderwerpen verder uit te diepen. Mocht u bereid zijn tot een interview, dan vernemen wij dit graag. Het interview zal ongeveer een uur duren en kan plaatsvinden op een locatie naar uw keuze. Als blijk van waardering ontvangt u van ons voor een interview een boekenbon ter waarde van €25,=.

Wij kunnen om anonimiteitsredenen uw contactgegevens niet achterhalen. In het geval dat u bereid bent tot een interview, verzoeken wij u uw naam en adresgegevens hieronder in te vullen. U bent dan niet meer anoniem. Als u wordt uitgekozen, nemen wij telefonisch of per email contact met u op.

Wij stellen uw deelname aan dit onderzoek enorm op prijs!

Bij voorbaat hartelijk dank.

Naam: ………………………………………………………………..

Adres: ………………………………………………………………

Telefoonnummer: ……………………………………………

E-mailadres: ……………………………………………………

Uw opmerkingen

Hebt u nog opmerkingen of aanvullingen na het invullen van deze vragenlijst, dan vernemen wij deze graag.

Einde

Hartelijk dank voor uw medewerking!

Voor vragen of opmerkingen naar aanleiding van dit onderzoek of deze vragenlijst kunt u contact opnemen met Drs. P.S.C. Kouwenhoven, arts-onderzoeker.
E-mail P.S.C.Kouwenhoven@umcutrecht.nl
Tel. 088-755 5125
