# Supplementary material for: Opinions about euthanasia and advanced dementia: a qualitative study among Dutch physicians and members of the general public
Source: BMC Med Ethics. 2015 Jan 28;16:7. doi: 10.1186/1472-6939-16-7 (PMC4350907; doi:10.1186/1472-6939-16-7)
Supplement: Supplementary file 2 — Additional file 2: Questionnaire for the general public. (PDF 57 KB) [file 12910_2014_328_MOESM2_ESM.pdf]

Inleiding

Deze vragenlijst gaat over ziekte en gezondheid. In het bijzonder wordt aandacht besteed aan medische zorg en behandelingen in de laatste levensfase. Wij willen graag weten hoe u hierover denkt, wat u hierover weet, en of u hier in uw omgeving ooit mee te maken hebt gehad.
Voor sommige mensen zijn medische zorg en behandelingen in de laatste levensfase onderwerpen waar zij liever niet teveel over nadenken. Toch stellen wij het zeer op prijs als u de tijd en moeite zou willen nemen om deze vragenlijst in te vullen.
Het onderzoek wordt uitgevoerd in opdracht van het Ministerie van Volksgezondheid, Welzijn en Sport. De resultaten van het onderzoek zullen worden gebruikt voor het beleid. Voor het verkrijgen van een betrouwbaar beeld is *uw* bijdrage aan het onderzoek erg belangrijk!

Het beantwoorden van de gehele vragenlijst zal ongeveer 25 minuten duren.

Wanneer u een vraag hebt beantwoord, kunt u niet meer terug in de vragenlijst.

Uw eigen ervaringen

Mensen komen op verschillende manieren in aanraking met thema's rond ziekte, gezondheid en medische zorg en behandelingen in de laatste levensfase. Voor sommige mensen spelen televisie, kranten, tijdschriften en internet hierbij een belangrijke rol.

1. Er zijn allerlei programma's op televisie die aandacht besteden aan medische zorg en behandelingen in de laatste levensfase. Kijkt u naar televisieprogramma's die hieraan aandacht besteden?
   [ ] Zelden of nooit
   [ ] Een of enkele keer per jaar
   [ ] Een of enkele keer per maand
   [ ] Een of enkele keer per week
   [ ] Dagelijks

2. Kranten, weekbladen en andere tijdschriften schrijven vaak over medische zorg en behandelingen in de laatste levensfase. Leest u artikelen die hierover gaan?
   [ ] Zelden of nooit
   [ ] Een of enkele keer per jaar
   [ ] Een of enkele keer per maand
   [ ] Een of enkele keer per week
   [ ] Dagelijks

3. Op internet wordt vaak geschreven over medische zorg en behandelingen in de laatste levensfase. Bezoekt u websites die hierover gaan?
   [ ] Zelden of nooit

[ ] Een of enkele keer per jaar

[ ] Een of enkele keer per maand

[ ] Een of enkele keer per week

[ ] Dagelijks

4. Hebt u wel eens behoefte aan meer informatie over onderwerpen zoals medische zorg en behandelingen in de laatste levensfase, euthanasie of wilsverklaringen?

[ ] Nooit

[ ] Zelden

[ ] Soms

[ ] Regelmatig

5. Welke informatiebronnen zou u raadplegen als u meer wilt weten over onderwerpen zoals medische zorg en behandelingen in de laatste levensfase, euthanasie of wilsverklaringen? *(meer dan een antwoord mogelijk)*

[ ] De televisie

[ ] Kranten of tijdschriften

[ ] Internet

[ ] Mijn huisarts

[ ] Mijn specialist

[ ] Familie / vrienden

[ ] Anders, namelijk….

[ ] Weet ik niet

In uw eigen leven bent u mogelijk ook in aanraking gekomen met vraagstukken over de laatste levensfase. Bijvoorbeeld doordat een van uw naasten, zoals een familielid, vriend of een andere belangrijke persoon in uw omgeving, ernstig ziek is of is geweest.

6.

a) Heeft *in de afgelopen 5 jaar* een van uw naasten een arts om *euthanasie gevraagd*?

[ ] Nee - > ga naar vraag 7

[ ] Ja

[ ] Weet ik niet

b) Hebt u *in de afgelopen 5 jaar* meegemaakt dat er bij een van uw naasten *euthanasie is uitgevoerd*?

[ ] Nee

[ ] Ja

[ ] Weet ik niet

c) Hebt u *in de afgelopen 5 jaar* meegemaakt dat een arts een verzoek om *euthanasie* van een van uw naasten heeft *afgewezen?*

[ ] Nee

[ ] Ja

[ ] Weet ik niet

d) Welke indruk hebt u van de manier waarop artsen in het algemeen met euthanasieverzoeken omgaan?

[ ] Artsen gaan serieus om met dergelijke verzoeken

[ ] Artsen wijzen dergelijke verzoeken zoveel mogelijk af

[ ] Weet ik niet

7. Is *in de afgelopen 5 jaar* een van uw naasten door een arts in *een diepe slaap gebracht tot aan het overlijden*?

[ ] Nee

[ ] Ja

[ ] Weet ik niet

We zijn ook geïnteresseerd in uw gezondheid en uw ideeën over medische zorg en behandelingen in de laatste levensfase.

8. Hebt u ooit een levensbedreigende ziekte gehad, of heeft u momenteel een levensbedreigende ziekte?

[ ] Nee

[ ] Ja, ik heb ooit een levensbedreigende ziekte gehad, maar nu niet meer

[ ] Ja, ik heb momenteel een levensbedreigende ziekte

9. Hebt u weleens over onderstaande onderwerpen nagedacht? -> indien allemaal Nee, ga naar vraag 13

	Nee	Ja
Medische behandelingen die ik wel of niet zou willen in mijn laatste levensfase	[ ]	[ ]
Of en in welke omstandigheden ik gereanimeerd zou willen worden	[ ]	[ ]
Of en in welke omstandigheden ik euthanasie zou willen	[ ]	[ ]
Wie voor mij beslist over medische behandelingen wanneer ik dit zelf niet meer kan	[ ]	[ ]

10. Spreekt u wel eens met *een van uw naasten* over een van deze onderwerpen?

[ ] Nooit

[ ] Zelden

[ ] Soms

[ ] Regelmatig

11. Spreekt u wel eens *met uw arts* over een van deze onderwerpen?
   [ ] Nooit
   [ ] Zelden
   [ ] Soms
   [ ] Regelmatig

12. Hebt u uw ideeën over de medische zorg en behandelingen die u wel of niet zou willen ontvangen in de laatste levensfase op papier vastgelegd?
   [ ] Nee -> ga naar vraag 13
   [ ] Ja

a) Kunt u aangeven wat u schriftelijk vastgelegd over medische zorg en behandelingen in uw laatste levensfase?

	Nee	Ja
De omstandigheden waarin ik bepaalde medische behandelingen niet meer wil	[ ]	[ ]
De omstandigheden waarin ik niet meer gereanimeerd wil worden	[ ]	[ ]
De omstandigheden waarin ik euthanasie wil	[ ]	[ ]
Wie voor mij beslissingen mag nemen als ik dat zelf niet meer kan	[ ]	[ ]
Andere ideeën; namelijk...	[ ]	[ ]

13. Hoeveel vertrouwen hebt u dat artsen uw wensen over medische zorg en behandelingen in de laatste levensfase zullen volgen?
   [ ] Geen enkel vertrouwen
   [ ] Niet veel vertrouwen
   [ ] Tamelijk veel vertrouwen
   [ ] Zeer veel vertrouwen
   [ ] Weet ik niet

14. Weet u hoe uw huisarts denkt over euthanasie?
   [ ] Nee, geen idee -> ga door naar vraag 15
   [ ] Ja, enigszins
   [ ] Ja

   a. Kunt u aangeven hoe uw huisarts denkt over euthanasie?
      [ ] Mijn huisarts staat open voor euthanasie
      [ ] Mijn huisarts is terughoudend met euthanasie
      [ ] Mijn huisarts is tegen euthanasie

Uw kennis

Er zijn verschillende soorten medische zorg en behandelingen mogelijk in de laatste levensfase. Vaak worden hierbij niet-alledaagse begrippen gebruikt. De volgende vragen gaan over of u *uit uw hoofd* weet wat deze begrippen inhouden. Het is daarom belangrijk dat u geen informatie opzoekt.

15. Weet u wat het begrip *''palliatieve zorg''* inhoudt?
    [ ] Nee, nooit van gehoord
    [ ] Nee, maar wel eens van gehoord
    [ ] Ja, enigszins
    [ ] Ja

16. Weet u wat het begrip *"palliatieve sedatie"* inhoudt?
    [ ] Nee, nooit van gehoord
    [ ] Nee, maar wel eens van gehoord
    [ ] Ja, enigszins
    [ ] Ja

17. Weet u wat het begrip *"euthanasie"* inhoudt?
    [ ] Nee, nooit van gehoord
    [ ] Nee, maar wel eens van gehoord
    [ ] Ja, enigszins
    [ ] Ja

18. Weet u wat een *"schriftelijke wilsverklaring"* is?
    [ ] Nee, nooit van gehoord
    [ ] Nee, maar wel eens van gehoord
    [ ] Ja, enigszins
    [ ] Ja

In wetgeving wordt ook aandacht besteed aan medische zorg en behandelingen in de laatste levensfase. Met de volgende vragen en stellingen willen we nagaan wat u *uit uw hoofd* wel en niet weet over deze wetgeving. Ook hier is het belangrijk dat u geen informatie opzoekt.

Euthanasie

In Nederland hebben we een euthanasiewet. Deze wet houdt in dat een arts niet strafbaar is als hij euthanasie of hulp bij zelfdoding toepast mits hij zich aan een aantal voorwaarden houdt.

19. Was u op de hoogte van het bestaan van deze wet?
    [ ] Nee
    [ ] Ja

We willen nagaan wat u *uit uw hoofd* weet over de regulering van euthanasie in Nederland. Ook als u niet op de hoogte was van het bestaan van de euthanasiewet, vragen wij u om de volgende vragen te beantwoorden.

20. De heer A denkt dat iedereen die dat wil recht heeft op euthanasie. De heer B denkt dat dit niet waar is. Wie heeft er *wettelijk gezien* gelijk?
    [ ] De heer A
    [ ] De heer B
    [ ] Weet ik niet

21. De heer C denkt dat euthanasie *alleen* mag worden uitgevoerd bij een patiënt die nog maar enkele weken te leven heeft. De heer D denkt dateuthanasie ook mag worden uitgevoerd bij patiënten met een langere levensverwachting. Wie heeft er *wettelijk gezien* gelijk?
    [ ] De heer C
    [ ] De heer D
    [ ] Weet ik niet

22. Mevrouw E denkt dat euthanasie *alleen* is toegestaan wanneer iemand ernstig lijdt aan een onbehandelbare ziekte. Mevrouw F denkt dat euthanasie ook is toegestaan bij iemand die niet meer verder wil leven, zonder dat hij of zij ernstig ziek is. Wie heeft er *wettelijk gezien* gelijk?
    [ ] Mevrouw E
    [ ] Mevrouw F
    [ ] Weet ik niet

23. Mevrouw G denkt dat euthanasie *alleen* is toegestaan als een patiënt daar zelf om vraagt. Mevrouw H denkt dat euthanasie ook zonder verzoek van de patiënt mogelijk is. Wie heeft er *wettelijk gezien* gelijk?
    [ ] Mevrouw G
    [ ] Mevrouw H
    [ ] Weet ik niet

Uw opvattingen

De volgende vragen gaan niet over wat u weet over de euthanasiewet, maar over wat *u zelf* vindt van dit onderwerp.

Wilt u aangeven in hoeverre u het eens bent met onderstaande stellingen?	Helemaal oneens	Oneens	Noch eens, noch oneens	Eens	Helemaal eens	Weet ik niet
24. Ik vind dat iedereen het recht moet hebben om euthanasie te krijgen als hij of zij dit wil.	☐	☐	☐	☐	☐	☐
25. Ik ben in alle gevallen tegen euthanasie.	☐	☐	☐	☐	☐	☐
26. Ik vind dat een arts alleen euthanasie mag uitvoeren bij iemand die nog maar enkele weken te leven heeft.	☐	☐	☐	☐	☐	☐
27. Ik vind dat euthanasie toegestaan moet zijn bij mensen die niet meer verder willen leven zonder dat zij lijden aan een ernstige ziekte.	☐	☐	☐	☐	☐	☐
28. Ik vind dat euthanasie alleen uitgevoerd mag worden wanneer een patiënt daar zelf om vraagt.	☐	☐	☐	☐	☐	☐
29. Ik vind dat ieder mens het recht heeft om zelf te beschikken over zijn eigen leven en dood.	☐	☐	☐	☐	☐	☐
30. Ik vind dat een arts er altijd alles aan moet doen een ongeneeslijk zieke patiënt in leven te houden.	☐	☐	☐	☐	☐	☐
31. Ik vind dat een arts moet stoppen met de behandeling als een patiënt daar om vraagt.	☐	☐	☐	☐	☐	☐

32. Ik vind dat hoogbejaarden medicijnen moeten kunnen krijgen waarmee zij, als zij dat willen, een einde aan hun leven kunnen maken.	☐	☐	☐	☐	☐		☐
33. Ik vind dat een arts beter kan beoordelen wat voor de patiënt het beste is, dan dat de patiënt dit zelf van te voren kan bedenken.	☐	☐	☐	☐	☐		☐
34. Ik vind dat een arts informatie voor de patiënt mag achterhouden, als hij denkt dat dit beter is voor de patiënt.	☐	☐	☐	☐	☐		☐

In de laatste levensfase worden vaak beslissingen genomen over de medische zorg en behandelingen. Sommige mensen vinden dat de arts deze beslissingen zou moeten nemen. Anderen vinden dat de patiënt deze beslissingen zou moeten nemen.

35. Kunt u aangeven welke beschrijving van besluitvorming over medische zorg en behandelingen in de laatste levensfase uw voorkeur heeft?

Ik vind dat een arts mij goed moet informeren, en dat…

[ ] … hij de beslissing moet nemen, op basis van wat hij denkt dat het beste voor mij is.
[ ] … wij samen de beslissing moeten nemen, als een gezamenlijk besluit.
[ ] … ik zelf de beslissing moet nemen, op basis van wat ik denk dat het beste voor mij is.

Als een patiënt ongeneeslijk ziek is en wil sterven kan hij de arts vragen hem hierbij te helpen. De arts kan de patiënt een dodelijk middel toedienen met een injectie. De patiënt kan ook zelf dit dodelijk middel innemen met een drankje, onder toezicht van de arts.

36. Als beide manieren van het toedienen van een dodelijk middel mogelijk zijn, welke van de onderstaande handelwijzen zou volgens u de voorkeur hebben?

[ ] De arts geeft de patiënt een dodelijke injectie
[ ] De patiënt drinkt het dodelijke drankje zelf op, onder toezicht van de arts
[ ] Geen voorkeur
[ ] Anders, namelijk: …

Uw mening over verschillende situaties

In het volgende deel van de vragenlijst worden u een aantal verschillende situaties voorgelegd. Dit zijn verzonnen situaties, die in werkelijkheid kunnen voorkomen. De vragen bij deze situaties gaan over wat u weet en wat uw mening is.

Per situatie zullen er drie vragen worden gesteld. De omschrijving van de situatie zal bij iedere vraag herhaald worden.

Mevrouw Jansen (69 jaar) heeft ongeneeslijke botkanker. Zij heeft waarschijnlijk nog enkele dagen te leven. Zij heeft ernstige pijnklachten en krijgt hiervoor morfine, maar dit helpt nog niet voldoende. Na overleg met mevrouw Jansen verhoogt de arts de dosis morfine om de pijn te verlichten. De pijn is hierna onder controle. Mevrouw Jansen overlijdt enkele dagen later.

Mevrouw Jansen (69 jaar) heeft ongeneeslijke botkanker. Zij heeft waarschijnlijk nog enkele dagen te leven. Zij heeft ernstige pijnklachten en krijgt hiervoor morfine, maar dit helpt nog niet voldoende. Na overleg met mevrouw Jansen verhoogt de arts de dosis morfine om de pijn te verlichten. De pijn is hierna onder controle. Mevrouw Jansen overlijdt binnen een dag.

Mevrouw Jansen (69 jaar) heeft ongeneeslijke botkanker. Zij heeft waarschijnlijk nog enkele dagen te leven. Zij heeft ernstige pijnklachten en krijgt hiervoor morfine, maar dit helpt nog niet voldoende. Na overleg met mevrouw Jansen verhoogt de arts de dosis morfine om het levenseinde te bespoedigen. Mevrouw Jansen overlijdt binnen een dag.

Mevrouw Jansen (69 jaar) heeft ongeneeslijke botkanker. Zij heeft waarschijnlijk nog enkele dagen te leven. Zij heeft ernstige pijnklachten en krijgt hiervoor morfine, maar dit helpt nog niet voldoende. Na overleg met mevrouw Jansen verhoogt de arts de dosis morfine om het levenseinde te bespoedigen. Mevrouw Jansen overlijdt enkele dagen later.

37. Vindt u persoonlijk het handelen van de arts in deze situatie juist?
    - [ ] Ja
    - [ ] Nee
    - [ ] Weet ik niet

Deze vraag gaat ook over de situatie van mevrouw Jansen. De omschrijving van de situatie wordt hier herhaald.
(*herhaling vignet*)

38. Hoe zou u het handelen van deze arts noemen?
    - [ ] Pijn- en/of symptoombestrijding
    - [ ] Palliatieve sedatie

[ ] Stoppen met behandeling

[ ] Euthanasie

[ ] Levensbeëindiging zonder verzoek van de patiënt

[ ] Anders, namelijk...

[ ] Weet ik niet

Deze vraag gaat ook over de situatie van mevrouw Jansen. De omschrijving van de situatie wordt hier herhaald.
(*herhaling vignet*)

39. Is het handelen van deze arts in Nederland toegestaan?

    [ ] Ja

    [ ] Nee

    [ ] Weet ik niet

De heer Avezaath is een man van 70 jaar oud met ongeneeslijke darmkanker. Hij heeft ernstige pijnklachten. De arts verwacht dat hij binnen een week zal overlijden. De morfine die hij krijgt helpt onvoldoende tegen de pijn. In overleg met de heer Avezaath besluit de arts om hem in diepe slaap te brengen tot het overlijden, zodat hij tot aan zijn overlijden geen last meer heeft van de pijn. Hij kan dan niet meer zelf eten of drinken en krijgt ook geen vocht of voeding toegediend. De arts dient de heer Avezaath slaapmiddelen toe, waarna hij al gauw in diepe slaap raakt en overlijdt na een week.

De heer Avezaath is een man van 70 jaar oud met ongeneeslijke darmkanker. Hij heeft ernstige pijnklachten. De arts verwacht dat hij binnen een maand zal overlijden. De morfine die hij krijgt helpt onvoldoende tegen de pijn. In overleg met de heer Avezaath besluit de arts om hem in diepe slaap te brengen tot het overlijden, zodat hij tot aan zijn overlijden geen last meer heeft van de pijn. Hij kan dan niet meer zelf eten of drinken en krijgt ook geen vocht of voeding toegediend. De arts dient de heer Avezaath slaapmiddelen toe, waarna hij al gauw in diepe slaap raakt en overlijdt na een week.

De heer Avezaath is een man van 70 jaar oud met ongeneeslijke darmkanker. Hij heeft ernstige pijnklachten. De arts verwacht dat hij binnen een maand zal overlijden. De morfine die hij krijgt helpt onvoldoende tegen de pijn. In overleg met de heer Avezaath besluit de arts om hem in diepe slaap te brengen tot het overlijden, zodat hij zo snel mogelijk zal overlijden. Hij kan dan niet meer zelf eten of drinken en krijgt ook geen vocht of voeding toegediend. De arts dient de heer Avezaath slaapmiddelen toe, waarna hij al gauw in diepe slaap raakt en overlijdt na een week.

40. Vindt u persoonlijk het handelen van de arts in deze situatie juist?

    [ ] Ja

    [ ] nee

    [ ] Weet ik niet

Deze vraag gaat ook over de situatie van de heer Avezaath. De omschrijving van de situatie wordt hier herhaald.
(*herhaling vignet*)

41. Hoe zou u het handelen van deze arts noemen?
    [ ] Pijn- en/of symptoombestrijding
    [ ] Palliatieve sedatie
    [ ] Stoppen met behandeling
    [ ] Euthanasie
    [ ] Levensbeëindiging zonder verzoek van de patiënt
    [ ] Anders, namelijk...
    [ ] Weet ik niet

Deze vraag gaat ook over de situatie van de heer Avezaath. De omschrijving van de situatie wordt hier herhaald.
(*herhaling vignet*)

42. Is het handelen van deze arts in Nederland toegestaan?
    [ ] Ja
    [ ] Nee
    [ ] Weet ik niet

Mevrouw de Vries is 72 jaar en heeft ongeneeslijke baarmoederhalskanker. Naar verwachting heeft mevrouw de Vries nog een week te leven. Ze ligt met een luidruchtige en onregelmatige ademhaling in bed. Volgens de arts heeft zij hier zelf geen last van. Ze reageert niet meer op haar omgeving. Haar familieleden geven aan dat zij het vreselijk vinden hun moeder zo in bed te zien liggen. Zij kunnen het niet langer aanzien. Na overleg met de familie brengt de arts mevrouw de Vries in diepe slaap. Mevrouw de Vries overlijdt een week later.

Mevrouw de Vries is 72 jaar en heeft ongeneeslijke baarmoederhalskanker. Naar verwachting heeft mevrouw de Vries nog een week te leven. Ze ligt met een luidruchtige en onregelmatige ademhaling in bed. Volgens de arts heeft zij hier zelf geen last van. Ze reageert niet meer op haar omgeving. Haar familieleden geven aan dat zij het vreselijk vinden hun moeder zo in bed te zien liggen. Zij kunnen het niet langer aanzien. Na overleg met de familie geeft de arts mevrouw de Vries een spuitje, waarna zij binnen enkele minuten overlijdt.

Mevrouw de Vries is 72 jaar en heeft ongeneeslijke baarmoederhalskanker. Naar verwachting heeft mevrouw de Vries nog een week te leven. Ze ligt met een luidruchtige en onregelmatige ademhaling in bed. Volgens de arts heeft zij hier zelf geen last van. Ze reageert niet meer op haar omgeving. Haar

familieleden geven aan dat hun moeder het vreselijk zou vinden zo in bed te moeten liggen. Zij zeggen dat zij deze situatie zelf nooit zo gewild zou hebben. Na overleg met de familie brengt de arts mevrouw de Vries in diepe slaap. Mevrouw de Vries overlijdt een week later.

Mevrouw de Vries is 72 jaar en heeft ongeneeslijke baarmoederhalskanker. Naar verwachting heeft mevrouw de Vries nog een week te leven. Ze ligt met een luidruchtige en onregelmatige ademhaling in bed. Volgens de arts heeft zij hier zelf geen last van. Ze reageert niet meer op haar omgeving. Haar familieleden geven aan dat hun moeder het vreselijk zou vinden zo in bed te moeten liggen. Zij zeggen dat zij deze situatie zelf nooit zo gewild zou hebben. Na overleg met de familie geeft de arts mevrouw de Vries een spuitje, waarna zij binnen enkele minuten overlijdt.

43. Vindt u persoonlijk het handelen van de arts in deze situatie juist?
    [ ] Ja
    [ ] Nee
    [ ] Weet ik niet

Deze vraag gaat ook over de situatie van mevrouw de Vries. De omschrijving van de situatie wordt hier herhaald.
(*herhaling vignet*)

44. Hoe zou u het handelen van deze arts noemen?
    [ ] Pijn- en/of symptoombestrijding
    [ ] Palliatieve sedatie
    [ ] Stoppen met behandeling
    [ ] Euthanasie
    [ ] Levensbeëindiging zonder verzoek van de patiënt
    [ ] Anders, namelijk...
    [ ] Weet ik niet

Deze vraag gaat ook over de situatie van mevrouw de Vries. De omschrijving van de situatie wordt hier herhaald.
(*herhaling vignet*)

45. Is het handelen van deze arts in Nederland toegestaan?
    [ ] Ja
    [ ] Nee
    [ ] Weet ik niet

De heer Raat is een man van 55 jaar oud die een zwaar auto-ongeluk heeft gehad. Hij heeft het overleefd, maar ligt sinds het ongeluk in coma in het ziekenhuis. Om in leven te blijven krijgt hij vocht en

voeding door een slangetje in zijn neus. Er is geen contact met hem mogelijk, hij reageert nergens op. De artsen zijn na uitgebreid overleg van mening dat de patiënt hoogstwaarschijnlijk niet meer zal ontwaken uit zijn coma. Zij besluiten te stoppen met het geven van vocht en voeding via het slangetje in zijn neus, waarna de heer Raat zal overlijden. Na een gesprek met de familie verwijdert de arts het slangetje uit zijn neus. De familie is het hier niet mee eens.

De heer Raat is een man van 55 jaar oud die een zwaar auto-ongeluk heeft gehad. Hij heeft het overleefd, maar ligt sinds het ongeluk in coma in het ziekenhuis. Om in leven te blijven ligt hij aan een beademingsapparaat. Er is geen contact met hem mogelijk, hij reageert nergens op. De artsen zijn na uitgebreid overleg van mening dat de patiënt hoogstwaarschijnlijk niet meer zal ontwaken uit zijn coma. Zij besluiten te stoppen met de beademing, waarna de heer Raat zal overlijden. Na een gesprek met de familie haalt de arts hem van de beademing. De familie is het hier niet mee eens.

De heer Raat is een man van 55 jaar oud die een zwaar auto-ongeluk heeft gehad. Hij heeft het overleefd, maar ligt sinds het ongeluk in coma in het ziekenhuis. Om in leven te blijven krijgt hij vocht en voeding door een slangetje in zijn neus. Er is geen contact met hem mogelijk, hij reageert nergens op. De artsen zijn na uitgebreid overleg van mening dat de patiënt hoogstwaarschijnlijk niet meer zal ontwaken uit zijn coma. Zij besluiten te stoppen met het geven van vocht en voeding via het slangetje in zijn neus, waarna de heer Raat zal overlijden. Na een gesprek met de familie verwijdert de arts het slangetje uit zijn neus. De familie is het hier mee eens.

De heer Raat is een man van 55 jaar oud die een zwaar auto-ongeluk heeft gehad. Hij heeft het overleefd, maar ligt sinds het ongeluk in coma in het ziekenhuis. Om in leven te blijven ligt hij aan een beademingsapparaat. Er is geen contact met hem mogelijk, hij reageert nergens op. De artsen zijn na uitgebreid overleg van mening dat de patiënt hoogstwaarschijnlijk niet meer zal ontwaken uit zijn coma. Zij besluiten te stoppen met de beademing, waarna de heer Raat zal overlijden. Na een gesprek haalt de arts hem van de beademing. De familie is het hier mee eens.

46. Vindt u persoonlijk het handelen van de arts in deze situatie juist?
    [ ] Ja
    [ ] Nee
    [ ] Weet ik niet

Deze vraag gaat ook over de situatie van de heer Raat. De omscrhijving van de situatie wordt hier herhaald.
(*herhaling vignet*)

47. Hoe zou u het handelen van deze arts noemen?
    [ ] Pijn- en/of symptoombestrijding
    [ ] Palliatieve sedatie

[ ] Stoppen met behandeling

[ ] Euthanasie

[ ] Levensbeëindiging zonder verzoek van de patiënt

[ ] Anders, namelijk...

[ ] Weet ik niet

Deze vraag gaat ook over de situatie van de heer Raat. De omschrijving van de situatie wordt hier herhaald.
(*herhaling vignet*)

48. Is het handelen van deze arts in Nederland toegestaan?

[ ] Ja

[ ] Nee

[ ] Weet ik niet

De heer Smit is 62 jaar oud en dement. Hij herkent zijn vrouw en kinderen niet meer, weigert te eten en trekt zich steeds meer terug. Er is geen communicatie meer met hem mogelijk over de behandeling. Kort voordat hij dement werd heeft hij een schriftelijke euthanasieverklaring opgesteld waarin hij heeft vastgelegd dat zijn leven beëindigd moet worden als hij dement zou zijn. De familie is het hier mee eens. De arts besluit te doen wat de heer Smit heeft gevraagd en voert de euthanasie uit.

De heer Smit is 62 jaar oud en dement. Hij herkent zijn vrouw en kinderen niet meer, weigert te eten en trekt zich steeds meer terug. Er is geen communicatie meer met hem mogelijk over de behandeling. Kort voordat hij dement werd heeft hij een schriftelijke euthanasieverklaring opgesteld waarin hij heeft vastgelegd dat zijn leven beëindigd moet worden als hij dement zou zijn. De familie is het hier niet mee eens. De arts besluit te doen wat de heer Smit heeft gevraagd en voert de euthanasie uit.

De heer Smit is 62 jaar oud en dement. Hij herkent zijn vrouw en kinderen niet meer, weigert te eten en trekt zich steeds meer terug. Er is geen communicatie meer met hem mogelijk over de behandeling. Kort voordat hij dement werd heeft hij een schriftelijke wilsverklaring opgesteld waarin hij heeft vastgelegd dat hij als hij dement zou zijn geen antibiotica meer wil krijgen voor een longontsteking. Hij vindt dat zijn leven op die manier niet onnodig lang gerekt wordt. De familie is het hier mee eens. De heer Smit krijgt longontsteking. De arts besluit te doen wat de heer Smit heeft gevraagd en geeft hem geen antibiotica, waarna hij overlijdt.

De heer Smit is 62 jaar oud en dement. Hij herkent zijn vrouw en kinderen niet meer, weigert te eten en trekt zich steeds meer terug. Er is geen communicatie meer met hem mogelijk over de behandeling. Kort voordat hij dement werd heeft hij een schriftelijke wilsverklaring opgesteld waarin hij heeft vastgelegd dat hij als hij dement zou zijn geen antibiotica meer wil krijgen voor een

longontsteking. Hij vindt dat zijn leven op die manier niet onnodig lang gerekt wordt. De familie is het hier niet mee eens. De heer Smit krijgt longontsteking. De arts besluit te doen wat de heer Smit heeft gevraagd en geeft hem geen antibiotica, waarna hij overlijdt.

49. Vindt u persoonlijk het handelen van de arts in deze situatie juist?
    [ ] Ja
    [ ] Nee
    [ ] Weet ik niet

Deze vraag gaat ook over de situatie van de heer Smit. De omschrijving van de situatie wordt hier herhaald.
(*herhaling vignet*)

50. Zou u zelf een dergelijke schriftelijke wilsverklaring willen hebben?
    [ ] Ja
    [ ] Nee
    [ ] Weet ik niet

Deze vraag gaat ook over de situatie van de heer Smit. De omschrijving van de situatie wordt hier herhaald.
(*herhaling vignet*)

51. Is het handelen van deze arts in Nederland toegestaan?
    [ ] Ja
    [ ] Nee
    [ ] Weet ik niet

Mevrouw de Jong (60 jaar) heeft borstkanker met uitzaaiingen. Zij heeft verschillende behandelingen ondergaan, maar haar ziekte is niet meer te genezen. Op dit moment heeft ze geen lichamelijke klachten. Zij voelt dat ze de regie over haar leven verliest en zij vindt dit heel erg. Zij heeft in haar werkzame leven altijd zelf de touwtjes in handen gehad. Zij geeft aan dit niet meer vol te houden. Ze vraagt haar huisarts om een drankje om een einde te kunnen maken aan haar leven. De huisarts besluit haar het drankje te geven.

Mevrouw de Jong (60 jaar) heeft borstkanker met uitzaaiingen. Zij heeft verschillende behandelingen ondergaan, maar haar ziekte is niet meer te genezen. Zij heeft ernstige pijn, die niet goed te verlichten is. Daarnaast voelt zij dat ze de regie over haar leven verliest en zij vindt dit heel erg. Zij heeft in haar werkzame leven altijd zelf de touwtjes in handen gehad. Zij geeft aan dit niet meer vol te houden. Ze vraagt haar huisarts om een drankje om een einde te kunnen maken aan haar leven. De huisarts besluit haar het drankje te geven.

De heer de Bruyn is 86 jaar. Vroeger was hij professor aan de universiteit. Toen had hij plezier in zijn leven. Nu is hij oud en veel van zijn vrienden zijn dood. Hij is nooit getrouwd en heeft geen kinderen. Hij voelt zich vaak eenzaam. Hij is in goede lichamelijke en geestelijke conditie. De heer de Bruyn kan nog jaren leven. Maar hij ziet daar erg tegenop. Hij wil liever dood zijn. Dat heeft hij ook al vele malen tegen zijn huisarts gezegd. De heer de Bruyn vraagt de huisarts een drankje om een einde te kunnen maken aan zijn leven. De huisarts besluit hem het drankje te geven.

Mevrouw Langezaal is van middelbare leeftijd. Ze is lichamelijk gezond, maar geestelijk ziek. Ze is al jaren zeer depressief en de behandeling van de psychiater heeft niet geholpen. Ze zegt regelmatig tegen haar artsen dat ze dood wil. Ze heeft al eens geprobeerd zelfmoord te plegen, maar dat is mislukt. Mevrouw Langezaal gaat naar haar psychiater en vraagt om een drankje waarmee ze een einde aan haar leven kan maken. De psychiater besluit haar het drankje te geven.

Mevrouw de Koning is 65 jaar. Zij heeft last van beginnende dementie en is af en toe vergeetachtig. Zij is erg bang voor wat komen gaat. Zij vreest het verdere geheugenverlies en het moment dat zij haar omgeving niet meer zal herkennen. Zij heeft meegemaakt dat haar moeder zwaar dement was en zij wil dit proces zelf absoluut niet meemaken. Mevrouw de Koning vraagt haar huisarts een drankje om een einde te kunnen maken aan haar leven. De huisarts besluit haar het drankje te geven.

52. Vindt u persoonlijk het handelen van de arts in deze situatie juist?
    [ ] Ja
    [ ] Nee
    [ ] Weet ik niet

53. Vindt u persoonlijk dat er in deze situatie sprake is van ondraaglijk lijden?
    [ ] Ja
    [ ] Nee
    [ ] Weet ik niet

54. Stel dat u zich in de situatie van de patiënt zou bevinden. Zou u uw arts om levensbeëindiging vragen?
    [ ] Ja
    [ ] Nee
    [ ] Weet ik niet

55. Is het handelen van deze arts in Nederland toegestaan?
    [ ] Ja
    [ ] Nee
    [ ] Weet ik niet

Tot slot

Hartelijk dank voor het invullen van deze vragenlijst. Om een aantal onderwerpen nog wat verder uit te diepen, zouden we graag een aantal personen persoonlijk willen interviewen over hun ervaringen en ideeën over medische zorg en behandelingen in de laatste levensfase. Dit interview zal ongeveer 1 uur duren en kan plaatsvinden op een locatie naar uw keuze. Graag horen we of u eventueel bereid bent om deel te nemen aan zo'n interview.
Als u bereid bent om deel te nemen én hiervoor door ons geselecteerd wordt, zullen wij contact met u opnemen om een afspraak te maken.
Als blijk van waardering krijgt u na het interview een boekenbon ter waarde van 25 euro.

56. Bent u bereid om eventueel deel te nemen aan een vervolginterview?
    [ ] Nee
    [ ] Ja

57. Hebt u nog opmerkingen of aanvullingen naar aanleiding van deze vragenlijst?

---------------------------- Einde ----------------------------------
