# Supplementary material for: Opinions about euthanasia and advanced dementia: a qualitative study among Dutch physicians and members of the general public
Source: BMC Med Ethics. 2015 Jan 28;16:7. doi: 10.1186/1472-6939-16-7 (PMC4350907; doi:10.1186/1472-6939-16-7)
Supplement: Supplementary file 3 — Additional file 3: Interview guide. (PDF 47 KB) [file 12910_2014_328_MOESM3_ESM.pdf]

Bijlage B 2.1 Leidraad bij de kwalitatieve interviews[1]

Toelichting (leidraad) bij de wijze waarop tekst is afgedrukt:

CAPS	aanduiding van onderdelen
**Vet**	de vraag zo stellen
Normaal	aspecten die in het betreffende onderdeel aan bod moeten zijn gekomen
Cursief	suggestie voor vraagstelling

INTRODUCTIE

Te noemen punten:

- Rol = onderzoeker (nb: evt achtergrondinformatie van de interviewer bewaren voor ná het interview!)
- Onderzoek in opdracht van Ministerie van Volksgezondheid
- Doel en achtergrond onderzoek: in kaart brengen hoe professionals en burgers denken over de manier waarop vormen van medisch handelen handelingen rond het levenseinde in Nederland zijn geregeld
- Herinnering vragenlijstonderzoek + motivatie interview: verdieping, gedachten, ervaringen.
- Duur: ca 1 uur
- Opzet: 'open' deel en 'deel ahv casuistiek
- Anonimiteit dataverwerking
- Toestemming opname

Voorbeeld (introductie):

> *Ik ben als onderzoeker betrokken bij een onderzoek naar medisch handelen rond het levenseinde. In opdracht van het Ministerie van Volksgezondheid willen wij in kaart brengen hoe professionals en burgers denken over de manier waarop allerlei handelingen rond het levenseinde in Nederland zijn geregeld. U heeft al meegedaan met ons onderzoek door het invullen van een vragenlijst. We wilden ook graag een interview met u afnemen, omdat je in een gesprek wat dieper kunt ingaan op dit ingewikkelde en soms ook gevoelige onderwerp. Wij zijn dus erg geïnteresseerd naar uw gedachten hierover. Ook als u zelf ervaringen heeft op dit vlak, zouden wij daar graag meer over willen horen.*
>
> *Zoals aangegeven zal het gesprek ongeveer een uur duren. We hebben het interview opgezet in een heel 'open' en een meer gestructureerd deel. In het tweede deel wil ik u een paar korte casusbeschrijvingen, of verhaaltjes voorleggen, en daarbij zou ik steeds een paar vragen willen stellen. Voor ik begin met het eerste, open gedeelte, wil ik u vragen of u er bezwaar tegen heeft als ik dit interview opneem. Dat werkt voor mij een stuk gemakkelijker, omdat ik dan niet steeds alles hoef op te schrijven. Achteraf zullen wij de opname uitschrijven. De verwerking van de opname en de gegevens zal helemaal anoniem zijn. Uw naam wordt dus niet aan het bestand gekoppeld en is achteraf niet meer te achterhalen.*
>
> *Heeft u op dit moment nog vragen? Dan wil ik nu de opname starten.*

OPEN GEDEELTE

- open vragen

[1] Oorspronkelijk is voor elke geïnterviewde groep (burgers; artsen; verpleegkundigen en verzorgenden) een aparte leidraad gehanteerd. Deze leidraden zijn hier tot één variant gecombineerd. Variaties per afzonderlijke respondentgroep zijn aangegeven in de tekst.

- geen goede of foute antwoorden
- gelegenheid tot vertellen van eigen ervaringen

EUTHANASIE

1. **Als ik het woord 'euthanasie' noem, wat roept dit dan voor gedachten bij u op?**

2. **Stel u raakt in gesprek met een buitenlandse toerist, en die vraagt u hoe dat nou zit met euthanasie in Nederland. Wat zou u hem dan vertellen?**
   (wat het is, of het hier mag, onder welke omstandigheden, of respondent het goed vindt zoals het is geregeld)

2.(artsen)
Stel u raakt in gesprek met een buitenlandse collega, en die vraagt u hoe dat nou zit met euthanasie in Nederland. Wat zou u hem dan vertellen?
(wat het is, of het hier mag, onder welke omstandigheden, of respondent het goed vindt zoals het is geregeld)

3. **Heeft u in uw omgeving wel eens meegemaakt dat iemand om euthanasie vroeg, of euthanasie kreeg?**
   a. Ja → naar 'ervaring'
   b. Nee → naar volgende onderdeel
   b.(verpleegkundigen en verzorgenden)
   Nee → reden? (nooit patiënt met verzoek meegemaakt, anders) → naar volgende onderdeel
   b.(artsen)
   Nee → reden? (nooit verzoek gehad, principe kwestie, anders) → naar volgende onderdeel

ERVARING

- Indien meerdere ervaringen: kies de ervaring die u zich het beste kunt herinneren

- Details van het verhaal
  (Kunt u vertellen wat er gebeurde? Wanneer speelde zich het af? alleen bij verpleegkundige en verzorgende: *Wat was uw rol daarin?)*

- Handelingen
  (welke handelingen werden verricht – doel: onderscheid staken behandeling, 'spuitje' waarna dood, morfine, in slaap brengen)?

- Rol en helderheid van regels
  (Had u de indruk dat de zorgverlener zich aan bepaalde regels hield? Zo ja, welke dan? Denk aan zorgvuldigheidseisen, meldingsplicht)
  (Verpleegkundigen en verzorgende: *Hielden de betrokken zorgverleners zich aan bepaalde regels? Had u de indruk dat die regels helder waren en goed toe te passen? Knelpunten?*)
  (artsen: *Heeft u zich aan bepaalde regels gehouden, Waren die regels voor u helder en goed toe te passen? Waren er knelpunten?*)

- Ondraaglijk en uitzichtloos lijden (alleen voor verpleegkundigen en verzorgenden en artsen
  (Waaruit bestond het lijden van de patiënt? Vond u het ondraaglijk en uitzichtloos? Doorvragen waarom wel/niet

- Wettelijk toegestaan
  (Hoe denkt u dat dit past binnen de regels die er in Nederland gelden? (denk

aan de zorgvuldigheidseisen, meldingsplicht)
(voor verpleegkundigen en verzorgenden en artsen: *Denkt u dat het volgens de wet was toegestaan?*)

- Persoonlijke waardering
(Hoe kijkt u terug op het hele gebeuren (goed/niet goed, positief/negatief → doorvragen)

- Goede dood
(Wat vindt u van de wijze waarop de 'patiënt' uiteindelijk is overleden? Goed, slecht, iets heel anders? → doorvragen)

PALLIATIEVE SEDATIE

1. Naast het begrip 'euthanasie' worden ook wel andere woorden gebruikt om aan te geven op wat voor manier iemand is overleden. Een voorbeeld van zo'n term is 'palliatieve sedatie'. Heeft u daar wel eens van gehoord?

a. Ja → **Wat roept dat begrip voor gedachten bij u op?** (naar 2)
b. Nee → naar 3

1. (verpleegkundigen en verzorgenden en artsen): **Als ik het woord 'palliatieve sedatie'noem; wat roept dit dan voor gedachten bij u op?**

2. (verpleegkundigen en verzorgenden en artsen): **Stel u raakt in gesprek met een buitenlandse toerist, en die vraagt u hoe dat nou zit met 'palliatieve sedatie' in Nederland. Wat zou u hem dan vertellen?**
(Wat het is; of het hier mag; onder welke omstandigheden, of respondent het goed vindt zoals het is geregeld)

2. (artsen) **Stel u raakt in gesprek met een buitenlandse collega, en die vraagt u hoe dat nou zit met 'palliatieve sedatie' in Nederland. Wat zou u hem dan vertellen?**
(Wat het is; of het hier mag; onder welke omstandigheden, of respondent het goed vindt zoals het is geregeld)

2. (Burgers)**Heeft u in uw naaste omgeving wel eens een geval van 'palliatieve sedatie' meegemaakt?**

a. Ja → naar 'ervaring'
b. Nee → naar 3

3. Heeft u in uw naaste omgeving wel eens een sterfbed meegemaakt waarbij een zorgverlener de 'patiënt' in diepe slaap bracht tot aan het overlijden?

a. Ja → naar 'ervaring'
b. Nee → naar 4

3. (verpleegkundigen en verzorgenden) **Heeft u als verpleegkundige/verzorgende wel een een patiënt gehad bij wie palliatieve sedatie is uitgevoerd?**
c. Ja → naar 'ervaring'
d. Nee → reden? → naar volgend onderdeel

3. (artsen) **Heeft u als arts wel eens een palliatieve seadtie uitgevoerd?**

e. Ja → naar 'ervaring'
f. Nee → reden? (onvoldoende expertise, geen aanleiding voor geweest, anders naar → naar volgende onderdeel

4. Heeft u in uw naaste omgeving wel eens een sterfbed meegemaakt, waarbij een zorgverlener een rol speelde bij de manier waarop iemand kwam te overlijden.

a. Ja → naar ‘ervaring’
b. Nee → naar volgende onderwerp

ERVARING

- Indien meerdere ervaringen: kies de ervaring die u zich het beste kunt herinneren

- Details van het verhaal
  (Kunt u vertellen wat er gebeurde? Wanneer speelde zich het af) Voor verpleegkundigen en verzorgenden: *wat was uw rol daarin?*)

- Handelingen
  (welke handelingen werden verricht en door wie – doel: onderscheid staken behandeling, ‘spuitje’ waarna dood, morfine, in slaap brengen)? (Bij verpleegkundigen en verzorgenden en artsen alleen: *welke handelingen werden verricht?*)

- Rol van regels
  (Had u de indruk dat de zorgverlener zich aan bepaalde regels hield? Zo ja, welke dan? Denk aan 2 weken termijn, indicatie refractaire symptomen)
  (Verpleegkundigen en verzorgenden: *Hielden de betrokken zorgverleners zich aan bepaalde regels/ Had u de indruk dat die regels helder waren en goed toe te passen? Waren er knelpunten?*
  (artsen: *Heeft u zich aan bepaalde regels gehouden? Waren die regels voor u helder en goed toe te passen? Waren er knelpunten* denk aan: 2 weken termijn, refractaire symptomen, middelengebruik)

(Verpleegkundigen en verzorgenden en artsen:
- Ondraaglijk en uitzichtloos lijden / refractaire symptomen

  (Waaruit bestond het lijden van de patiënt/ Vond u het ondraaglijk en uitzichtloos? doorvragen waarom wel/niet)

- Wettelijk toegestaan (alleen voor burgers)
  (Hoe denkt u dat dit past binnen de regels die er in Nederland gelden? (denk aan de zorgvuldigheidseisen, meldingsplicht)

- Persoonlijke waardering
  (Hoe kijkt u terug op het hele gebeuren (goed/niet goed, positief/negatief → doorvragen)

- Goede dood
  (Wat vindt u van de wijze waarop de ‘patiënt’ uiteindelijk is overleden? Goed, slecht, iets heel anders? → doorvragen)

VERHOUDING EUTHANASIE - PALLIATIEVE SEDATIE (of andere door de respondent ingebrachte MHL)

(INDIEN NOG NIET TER SPRAKE GEKOMEN)

- Gepercipieerde verschillen / overeenkomsten euthanasie en palliatieve sedatie (alleen voor burgers: of een andere door de respondent ingebrachte vorm van medisch handelen rond het levenseinde)

  (We hebben het gehad over euthanasie en palliatieve sedatie (alleen voor burgers: een andere door de respondent ingebrachte vorm van medisch handelen rond het levenseinde). Hoe ziet u de verhouding tussen deze twee handelingen. Zijn dit voor u verschillende dingen, ziet u overeenkomsten?

  Indien een respondent hier op ingaat, kun je letten op verschil/overeenkomst mbt:
    - De handeling (doet de dokter iets heel anders?)
    - Het effect (gaat de patient eraan dood?)
    - Mate waarin het is toegestaan / geregeld
    - De morele beoordeling (vind de respondent beide ‘goed’, 1 beter dan ander? Waarom dan?

DEEL 2: CASUÏSTIEK

- er volgen nu drie korte casus (teksten voorleggen en laten lezen)
- eerder gebruikt in vragenlijst
- steeds enkele bijbehorende vragen
- geen goede of foute antwoorden

**CASUS 1**
De heer Avezaath is een man van 70 jaar. Hij heeft ongeneeslijke darmkanker met uitzaaiingen. Hij heeft ernstige pijnklachten. De arts verwacht dat hij binnen een maand zal overlijden. De morfine die hij krijgt helpt onvoldoende tegen de pijn. In overleg met de heer Avezaath besluit de arts om hem in diepe slaap te brengen, zodat hij tot aan zijn overlijden geen last meer heeft van de pijn. Hij kan dan niet meer zelf eten en drinken en krijgt ook geen vocht of voeding toegediend. De arts dient de heer Avezaath slaapmiddelen toe, waarna hij al gauw in diepe slaap raakt en overlijdt na een week.

Vraag:

1. **Wat zijn uw gedachten bij het lezen van deze casus?**

AANDACHTSPUNTEN:

- Persoonlijke moreel oordeel
Vindt u persoonlijk het handelen van de arts in deze situatie juist? (doorvragen waarom)

- Wettelijk toegestaan?
Is het handelen van deze arts in Nederland toegestaan? (doorvragen waarom)

- Overeenstemming/discrepantie moreel oordeel/wettelijk toegestaan
Is er sprake van kennis? Lopen wet en eigen opvatting parallel? Zo niet in welke richting wijkt is eigen opvatting anders?

- Hoe benoemt de respondent het handelen?
Hoe zou u het handelen van deze arts noemen (doorvragen waarom keuze voor bepaalde term)?

2. Aanbrengen variatie (indien nog niet ter sprake gekomen)

Stel dat de arts verwacht dat de heer Avezaath nog maar een week te leven zou hebben. Zou u dan anders over de casus denken?

Houd opnieuw bovenstaande aspecten in gedachten

CASUS 2

Mevrouw De Jong (60 jaar) heeft borstkanker met uitzaaiingen. Zij heeft verschillende behandelingen ondergaan, maar haar ziekte is niet meer te genezen. Op dit moment heeft ze geen lichamelijke klachten. Zij voelt dat ze de regie over haar leven verliest en zij vindt dit heel erg. Zij heeft in haar werkzame leven altijd zelf de touwtjes in handen gehad. Zij geeft aan dit niet meer vol te houden. Ze vraagt haar huisarts om een drankje om een einde te kunnen maken aan haar leven. De huisarts besluit haar het drankje te geven.

Vraag:

1. **Wat zijn uw gedachten bij het lezen van deze casus?**

   AANDACHTSPUNTEN:

   - Persoonlijke moreel oordeel
     Vindt u persoonlijk het handelen van de arts in deze situatie juist? (doorvragen waarom)

   - Wettelijk toegestaan?
     Is het handelen van deze arts in Nederland toegestaan? (doorvragen waarom)

   - Overeenstemming/discrepantie moreel oordeel/wettelijk toegestaan
     Is er sprake van kennis? Lopen wet en eigen opvatting parallel? Zo niet in welke richting is eigen opvatting anders?

   - Ondraaglijk (en uitzichtloos) lijden
     Indien de respondent niets uit zichzelf zegt over het lijden van de vrouw, kun je expliciet vragen of de respondent vindt dat hier sprake is van ondraaglijk lijden. (+doorvragen: waarom wel/niet)

2. Aanbrengen variatie (indien nog niet ter sprake gekomen)

   Stel dat mevrouw De Jong wél ernstige lichamelijke klachten zou hebben die de arts niet goed onder controle krijgt met medicatie. Zou u dan anders over de casus denken.

   Houd opnieuw bovenstaande aspecten in gedachten, eventueel lichamelijke klachten specificeren als ‘ernstige pijn’

CASUS 3

De heer Smit is 62 jaar oud en dement. Hij herkent zijn vrouw en kinderen niet meer, weigert te eten en trekt zich steeds meer terug. Er is geen communicatie meer met hem mogelijk over de behandeling. Kort voordat hij dement werd heeft hij een schriftelijke wilsverklaring opgesteld waarin hij heeft vastgelegd dat hij als hij dement zou zijn geen antibiotica meer wil krijgen voor een longontsteking. Hij vindt dat zijn leven op die manier onnodig lang gerekt wordt. De familie is het hier niet mee eens. De arts besluit te doen wat de heer Smit heeft gevraagd en hem bij een longontsteking geen antibiotica te geven, waarna hij overlijdt.

Vraag:

1. Wat zijn uw gedachten bij het lezen van deze casus?

AANDACHTSPUNTEN:

- Persoonlijke moreel oordeel
  Vindt u persoonlijk het handelen van de arts in deze situatie juist? (doorvragen waarom)

- Wettelijk toegestaan?
  Is het handelen van deze arts in Nederland toegestaan? (doorvragen waarom)

- Overeenstemming/discrepantie moreel oordeel/wettelijk toegestaan
  Is er sprake van kennis? Lopen wet en eigen opvatting parallel? Zo niet in welke richting is eigen opvatting anders?

2. Aanbrengen variatie (indien nog niet ter sprake gekomen)

A. **Stel dat de familie het wel eens is met de verklaring van Meneer Smit. Zou u dan anders over de casus denken?**

B. **Stel dat meneer Smit een andere verklaring zou hebben, waarin is vastgelegd dat hij wil dat zijn leven beëindigd wordt als hij dement zou zijn. Zou u dan anders over de casus denken?** (NB: familie is het eens met deze verklaring)

Houd opnieuw bovenstaande aspecten in gedachten

---

3. Heeft u zelf wel eens een situatie meegemaakt waarin een verklaring als die van meneer Smit een rol speelde?
   a. Ja → situatie uitvragen, focus op rol vd wilsverklaring in verloop
   b. Nee → naar 4

3. (verpleegkundigen en verzorgenden en artsen)
   Heeft u als verpleegkundige/ verzorgende/arts wel eens te maken gehad met een patiënt die een schriftelijke wilsverklaring had?
   a. Indien ja, naar 4
   b. indien nee, einde interview

4. Heeft u zelf een schriftelijke wilsverklaring of wel eens overwogen er een op te stellen?
   a. Ja → naar 5
   b. Nee → naar 6

4. Verpleegkundigen en verzorgende
   Uitvragen ervaring. Aandachtspunten:
   - Type wilsverklaring
   - Heeft die wilsverklaring een rol gespeeld in besluitvorming over een -behandeling?
   - Rol van de familie/naasten in eventuele besluitvorming

4. Artsen
   Uitvragen ervaring. Aandachtspunten:
   - Type wilsverklaring
   - Rol van de wilsverklaring in eventuele besluitvorming
   - Rol van familie/naasten in eventuele besluitvorming

**5.** Aandachtspunten:<br>- Type wilsverklaring<br>- Motief voor maken van wilsverklaring<br>- Zijn naasten op de hoogte van het bestaan vd verklaring?<br>- Is de eigen huisarts op de hoogte van het bestaan vd verklaring?	**6.** Aandachtspunten<br>- nooit aan gedacht, of bewuste keuze?

AFRONDING INTERVIEW

Denk aan de volgende punten:

- bedank de respondent voor deelname

- vraag hoe de respondent vond dat het interview verliep (open deel; casuïsitek; helderheid van de vragen; etc.). Noteer zo nodig eventuele klachten, bezwaren of onduidelijkheden, en geef deze bij verzenden van het geluidsbestand door aan BvdV.

- heeft de respondent verder nog vragen? Voor vragen over euthanasie en/of palliatieve sedatie verwijs je respondenten naar informatie die de overheid beschikbaar heeft gemaakt op het internet:

  http://www.rijksoverheid.nl/onderwerpen/euthanasie/vraag-en-antwoord/wie-kan-om-euthanasie-vragen.html

  http://www.rijksoverheid.nl/onderwerpen/euthanasie/palliatieve-sedatie-is-medische-handeling

- bedank de respondent nogmaals, en vergeet niet de **boekenbon** te overhandigen.

- laat de respondent ten slotte weten dat wij hem/haar indien gewenst op de hoogte kunnen brengen wanneer de resultaten van het onderzoek worden gepubliceerd. Indien de respondent dat wenst: noteer naam en contactgegevens (bij voorkeur emailadres), en geef dit bij het verzenden van de opname door aan BvdV.
