# Supplementary material for: Opinions about euthanasia and advanced dementia: a qualitative study among Dutch physicians and members of the general public
Source: BMC Med Ethics. 2015 Jan 28;16:7. doi: 10.1186/1472-6939-16-7 (PMC4350907; doi:10.1186/1472-6939-16-7)
Supplement: Supplementary file 4 — Additional file 4: Translated vignette and questions. (DOCX 14 KB) [file 12910_2014_328_MOESM4_ESM.docx]

**Translation of the vignette used in the survey and the face-to-face interview**

**MS: 3520812561248174, entitled Opinions of the Dutch general public and health care professionals about euthanasia and advanced dementia: a mixed method study.**

*Mr Smit is 62 years old and suffering from dementia. He doesn't recognise his wife and children anymore, refuses to eat and withdrawals into himself more and more. It is no longer possible to communicate with him about his treatment. Shortly before he became demented, he drafted an advance directive with a euthanasia request in case of dementia. His family agrees. The physician decides to honour his patient’s advance directive and performs euthanasia.*

The survey questions which we refer to in our article were:

Q1 Do you personally agree with the physician’s act?

Q2 Do you think that euthanasia is allowed in this case, in the Netherlands?

With regard to the topic list for the interviews: during the interview, the vignette of Mr. Smit was again presented to the respondent. Subsequently, the following questions were asked:

1. What are your thoughts when you read this case description?

2. Do you personally agree with the physician’s act in this case? Why?

3. Is the physicians act allowed in the Netherlands?

The interview guide requires the interviewer to discuss discrepancies and convergence between the respondent’s answer to the moral and the legal question
